# Metal homeostasis as a therapeutic lever: advancing metalloimmunology to remodel the tumor microenvironment and enhance cancer immunotherapy

**DOI:** 10.7150/thno.121988

**Published:** 2026-01-01

**Authors:** Xin Chen, Qi Wang, Hongxia Zhang, Ye Wang, Haiying Zhu

**Affiliations:** 1 Department of Cell Biology, Naval Medical University (Second Military Medical University), Shanghai 200433, China.; 2 Department of Urology, Chinese PLA General Hospital, Beijing 100853, China.

**Keywords:** metal homeostasis, tumor microenvironment, nanomedicine, metalloimmunotherapy

## Abstract

Targeting the dysregulation of essential metal homeostasis represents a rapidly evolving frontier in cancer immunotherapy. The tumor microenvironment (TME) is a complex immunosuppressive ecosystem comprising tumor cells, immune cells, stromal components, extracellular matrix, and diverse cytokines/chemokines, characterized by hypoxia, acidosis, elevated redox stress, and metabolic dysregulation that drive tumor progression and immunotherapy resistance. Crucially, dysregulated homeostasis of essential metals (e.g., Cu, Fe, Zn, Mg, Mn, Ca, Cr, Na, K) pervades the TME, directly promoting tumorigenesis through oncogenic pathway activation and aberrant energy metabolism while facilitating immune evasion, amplifying immunosuppression, and undermining cancer immunotherapies. In response, recent strategies have focused on leveraging metalloimmunology to reprogram the TME via: (1) activation of innate/adaptive immunity, (2) disruption of tumor metabolism, (3) induction of programmed cell death, and (4) triggering of immunogenic cell death (ICD). These approaches synergize with existing immunotherapies to enhance efficacy, aided by nanotechnology-enabled precision delivery of metal-based agents. In conclusion, by mastering the intricate interplay between metal ions and the immunosuppressive TME, these strategies hold immense potential to remodel the TME, reinvigorate anti-tumor immunity, and ultimately enhance the efficacy of next-generation cancer immunotherapies. This review presents metalloimmunology as an integrative paradigm connecting metal biology, tumor immunology, and nanotechnology, providing a transformative outlook for immunotherapy.

## Introduction

Cancer immunotherapy, as the fourth major cancer treatment modality following surgery, radiotherapy, and chemotherapy, achieves targeted tumor killing by activating or rebuilding the patient's own immune system, thus effectively overcoming the inherent limitations of traditional therapies such as non-selective killing and severe adverse reactions. Clinical evidence highlights the transformative impact of three principal immunotherapeutic approaches—immune checkpoint blockade (ICB), cellular therapies such as chimeric antigen receptor T-cell (CAR-T) therapy, and tumor vaccines—in managing aggressive malignancies. These immunotherapies have shown remarkable efficacy against diverse cancers, due to their unique strengths in precision targeting and broad clinical applicability 1, 2. Nevertheless, current data reveals that over 70% of patients still do not benefit from ICB therapy 3, primarily due to immune tolerance, immunosuppressive TME, and off-target toxicities. Among these factors, the immunosuppressive TME stands as a critical barrier to immunotherapy efficacy. Consequently, strategies to remodel the TME and enhance the functionality of effector immune cells have emerged as pivotal research directions for improving the clinical outcomes of existing therapies.

The TME consists of tumor cells, surrounding immune cells, cancer-associated fibroblasts (CAFs), extracellular matrix, microvasculature, and diverse cytokines and chemokines. It constitutes a complex system characterized by hypoxia, low pH, elevated glutathione (GSH), reactive oxygen species (ROS) levels and adenosine triphosphate (ATP), and dysregulated enzyme expression. Recent studies have revealed that metal elements are also critical components of the TME, and that disruptions in metal homeostasis are one of its defining features.

The biological effects of metals have been recognized for millennia. Copper compounds were used for disinfection in ancient Egypt, and metal-based cancer treatments were recorded in the *Compendium of Materia Medica*. These empirical uses predate the discovery of underlying molecular mechanisms. In the 1960s, scientists discovered that cisplatin exerts antitumor effects by forming crosslinks with DNA guanine bases to block tumor cell replication, marking the advent of the "metal-based chemotherapy" era 4. Since then, metals have not only been utilized for anti-inflammatory and antibacterial purposes but also as drug agents for targeted delivery or energy-converting components in nanoparticle systems for cancer treatment 5, 6. Current research reveals that metal ions, as essential components of life, play critical roles in enzyme catalysis, electron transfer, signal transduction, and redox homeostasis. Consequently, dysregulation of essential metal can lead to functional abnormalities in tissues and organs 7. The immune system is particularly sensitive to such dysregulation. The immunomodulatory functions of metal ions include but are not limited to the following: (1) Acting as second messengers or enzyme cofactors to initiate immune cell activation, (2) Influencing immune cell differentiation and polarization, and (3) Regulating immune cell metabolism and energy supply 8. Thus, metal ions serve as pivotal "molecular switches" and "metabolic hubs" within the functional networks of immune cells.

Given these findings, the correlation between metal homeostasis in the TME and tumorigenesis, as well as its therapeutic applications, has attracted growing interest. Studies reveal that metals and their biomaterial derivatives in the TME can remodel the immunosuppressive TME through multiple mechanisms: inhibiting the activation of tumor-associated immunosuppressive cells, promoting the activation of tumor-suppressive immune cells 9, targeting tumor stroma and angiogenesis 10, and reversing tissue hypoxia and acidosis 11.

Recent advances in the integration of nanotechnology and metalloimmunology have rapidly expanded the development of metal-based nanomaterials. Metal-based nanomaterials are considered ideal therapeutic carriers due to their unique physical properties, enabling accurate tumor targeting and accumulation as well as stimuli-responsive drug release. Several current studies reveal that combining metal-based nanomaterials with immunotherapies can break through immunosuppressive barriers of the TME, establish long-lasting anti-tumor immune memory, and synergistically enhance therapeutic efficacy 12, 13.

In this review, we present a transformative perspective by establishing metalloimmunology as an integrative paradigm that bridges metal biology, tumor immunology, and therapeutic nanotechnology. Moving beyond a mere summary of individual metal effects, we construct a comprehensive framework illustrating how dysregulated homeostasis of essential metals such as copper, iron, zinc, and magnesium orchestrates immunosuppression within the TME. Importantly, we systematically explore nano-enabled therapeutic strategies—including engineered ion chelators, ionophores, and multimodal nanoplatforms—that enable precise spatiotemporal modulation of metal biology to remodel the TME and overcome resistance to immunotherapy. We further highlight cutting-edge applications where nanomaterials are designed not only for targeted metal delivery but also to synergistically induce ICD and amplify the efficacy of ICB, CAR-T cell therapy, and vaccines. By incorporating emerging insights into metalloproteins and metal crosstalk and extending the discussion to less-explored metals such as manganese and calcium, this review charts a forward-looking roadmap for precision metalloimmunotherapy, thereby defining a novel and rapidly evolving frontier in cancer treatment.

## 1. Copper

Clinical evidence strongly links dysregulated copper metabolism to malignant progression. Elevated copper and ceruloplasmin levels in serum and tumors, which are observed in hepatocellular carcinoma (HCC) 14, breast 15, lung 16, and hematological malignancies 17, are consistently associated with poor prognosis 18.

### 1.1 Mechanisms of copper homeostasis dysregulation driving immunosuppressive TME

Mechanistically, copper exerts paradoxical tumor-modulating effects. On the one hand, it promotes oncogenesis by enhancing mitochondrial metabolism, MAPK/ERK pathway activation, angiogenesis, immune evasion, and context-dependent cuproptosis induction. On the other hand, elevated copper suppresses tumors by triggering ICD and oxidative stress 19. Notably, high cuproptosis signatures are closely linked immunosuppressive TME landscapes formation.

#### 1.1.1 Copper homeostasis dysregulation drives tumor immune escape

**Figure 1:**
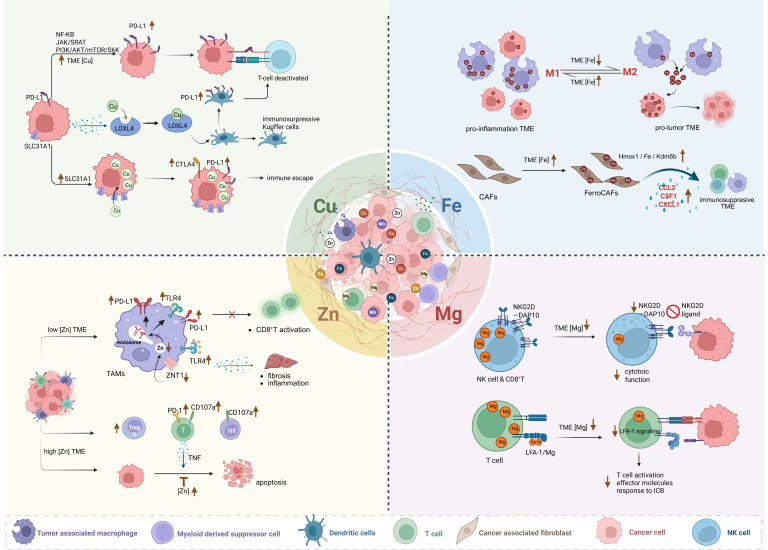
The representative mechanisms of four essential metal homeostasis imbalance within the TME driving the immunosuppressive microenvironment. Created in https://BioRender.com.

Dysregulated copper homeostasis in the TME arises from concurrent dysregulation of metabolic fluxes, copper-binding protein dysfunction, and defective transporter machinery. Copper imbalance within the TME facilitates tumor immune escape primarily by modulating immune checkpoint networks, thereby weakening immunotherapeutic efficacy **(Figure 1)**. Mechanistically, elevated TME copper levels upregulate programmed death-ligand 1 (PD-L1) expression on cancer cells via NF-κB, JAK/STAT, and PI3K/Akt/mTOR pathways 20. PD-L1 binding to PD-1 on T cells directly suppresses their antitumor function. Furthermore, HCC cells utilize copper-dependent lysyl oxidase-like 4 (LOXL4) to polarize Kupffer cells toward an immunosuppressive phenotype and upregulate PD-L1, synergistically inhibiting CD8⁺ T cells 21. Besides, the copper transporter SLC31A1 is overexpressed in multiple cancers and correlates with poor prognosis 22. It shows strong positive correlation with PD-L1/CTLA-4 expression 23, demonstrating that copper accumulation actively shapes an immune-evasive microenvironment. Together, these findings indicate that copper homeostasis dysregulation critically imbalances immune checkpoint networks, making it a promising target for enhancing immunotherapy.

From the perspective of cellular interactions within the TME, the flow of copper between tumor cells and immune cells may alter the anti-tumor function of immune cells. For example, neuroblastoma cells drive tumor progression by excessively taking up copper. This sequestering behavior leads to depletion of copper in the TME, thereby suppressing the function of immune cells, particularly neutrophils 24.

Copper further drives vascular-immune crosstalk in the TME. Neovascular endothelial tip cells establish an immunosuppressive niche by inducing T cell exhaustion 25, while copper directly potentiates pro-angiogenic factors (e.g., vascular endothelial growth factor (VEGF), FGF) through enhanced ligand-receptor binding 26. Mechanistically, the copper chaperone antioxidant 1 copper chaperone (ATOX1) activates PDGF signaling to drive smooth muscle migration and neointimal remodeling 27, and copper-mediated hypoxia-inducible factor-1α (HIF-1α) stabilization 28, 29 along with NF-κB activation upregulates VEGF expression 30. This coordination of vascular induction and immune suppression reveals novel therapeutic vulnerabilities in the copper-modulated TME.

#### 1.1.2 High cuproptosis signatures are closely associated with the formation of the immunosuppressive TME

Although copper accumulation triggers cuproptosis 31, tumor cells in hypoxic and copper-rich TME develop resistance to cuproptosis through copper binding to HIF-1α, which inhibits ubiquitin-mediated degradation of HIF-1α and amplifies hypoxia. This upregulating metallothionein 2A (MT2A) and pyruvate dehydrogenase kinase 1/3 (PDK1/3) while suppressing dihydrolipoamide S-acetyltransferase (DLAT) expression. Consequently, MT2A sequesters mitochondrial copper, conferring cuproptosis resistance and enabling tumor survival 32.

**Figure 2:**
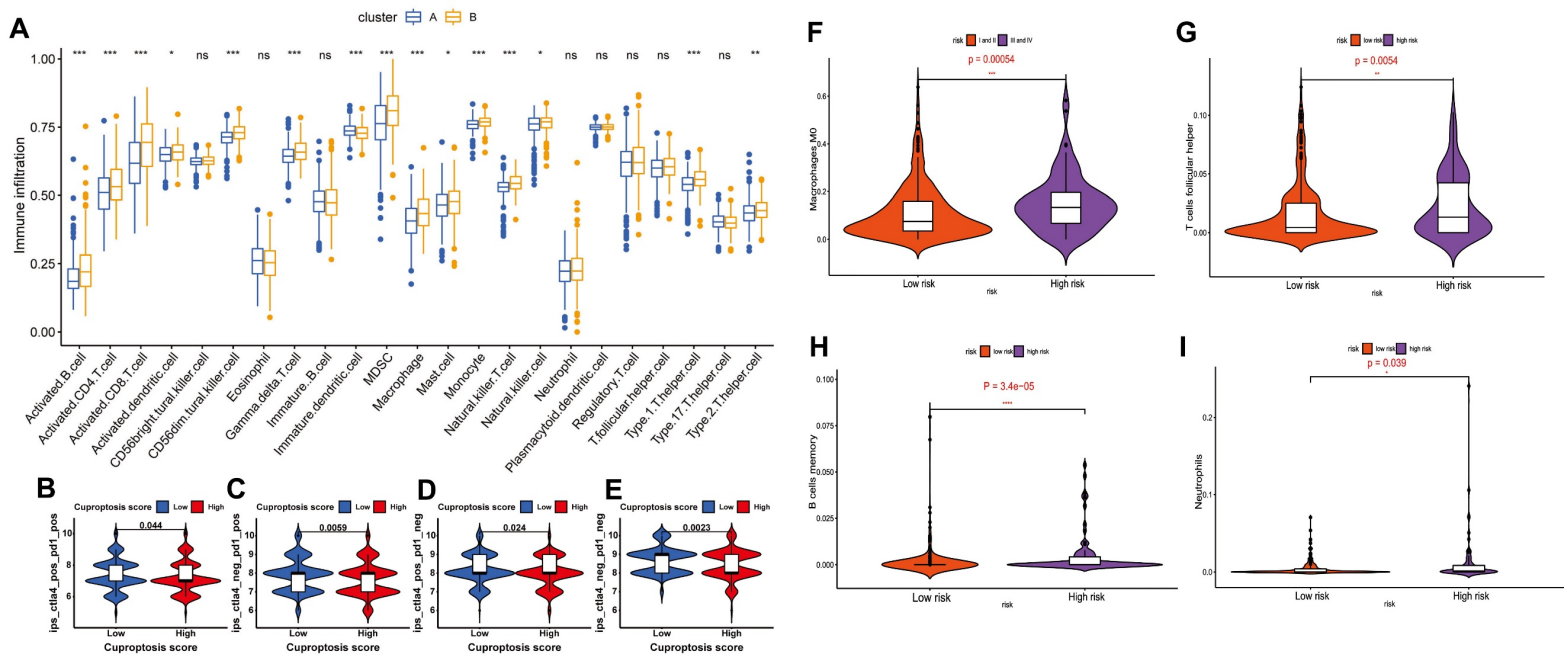
**Cuproptosis and ferroptosis status affects the tumor immune microenvironment and the efficacy of immunotherapy. A)** Tumor immune microenvironment analysis of two subtypes where cluster A has a better prognosis and shows higher expression of seven cuproptosis-promoting genes (FDX1, LIAS, LIPT1, DLD, DLAT, PDHA1 and PDHB) than cluster B (p <0.001). **B-E)** Analysis of the efficacy of 4 ICBs based on Cuproptosis score grouping predicted by IPS scores. ctla4_pos_pd1_pos **(B)**, ctla4_neg_pd1_pos **(C)**, ctla4_pos_pd1_neg **(D)** and ctla4_neg_pd1_neg **(E)**; IPS, immune cell proportion score; pos, positive; neg, negative. Adapted from ref.^36^. Copyright 2022 Zhang, Chen, Fang, Tai, Chen and Cao. Licensed under CC BY 4.0. **F-I)** The landscape of immune infiltration in patients with HCC in the high-risk and low-risk ferroptosis groups. Adapted from ref.^70^. Licensed under CC BY 4.0. Copyright 2020 The Author(s).

In addition to tumor cells evolving resistance to cuproptosis, high cuproptosis signatures in the TME are closely linked to the immunosuppressive TME as evidenced by three key observations: First, elevated cuproptosis-related genes (e.g., SLC31A1, FDX1) positively correlate with immunosuppressive cytokines, cells and immune checkpoint molecules while predicting poor prognosis. Second, in gliomas, elevated SLC31A1 expression is associated with M2 macrophage enrichment and poor prognosis but inversely links to anti-tumor immune cells (plasmacytoid dendritic cells (DCs), CD56ᵇʳ^i^ᵍʰᵗ NK cells, CD8⁺ T cells) 23, 33. Third, multi-omics models confirm that high cuproptosis risk signatures strongly predict immunosuppressive TME profiles and ICB therapy failure 34-37** (Figure 2A-E)**. These findings establish cuproptosis-related genes as biomarkers for predicting ICB efficacy and guiding personalized immunotherapy. Given these understandings, patients with tumors characterized by high cuproptosis signatures and immunosuppressive TME—which typically respond poorly to immunotherapy—may benefit from therapeutic strategies that induce cuproptosis to inhibit tumor progression.

### 1.2 Strategies targeting copper homeostasis dysregulation for cancer treatment

**Figure 3:**
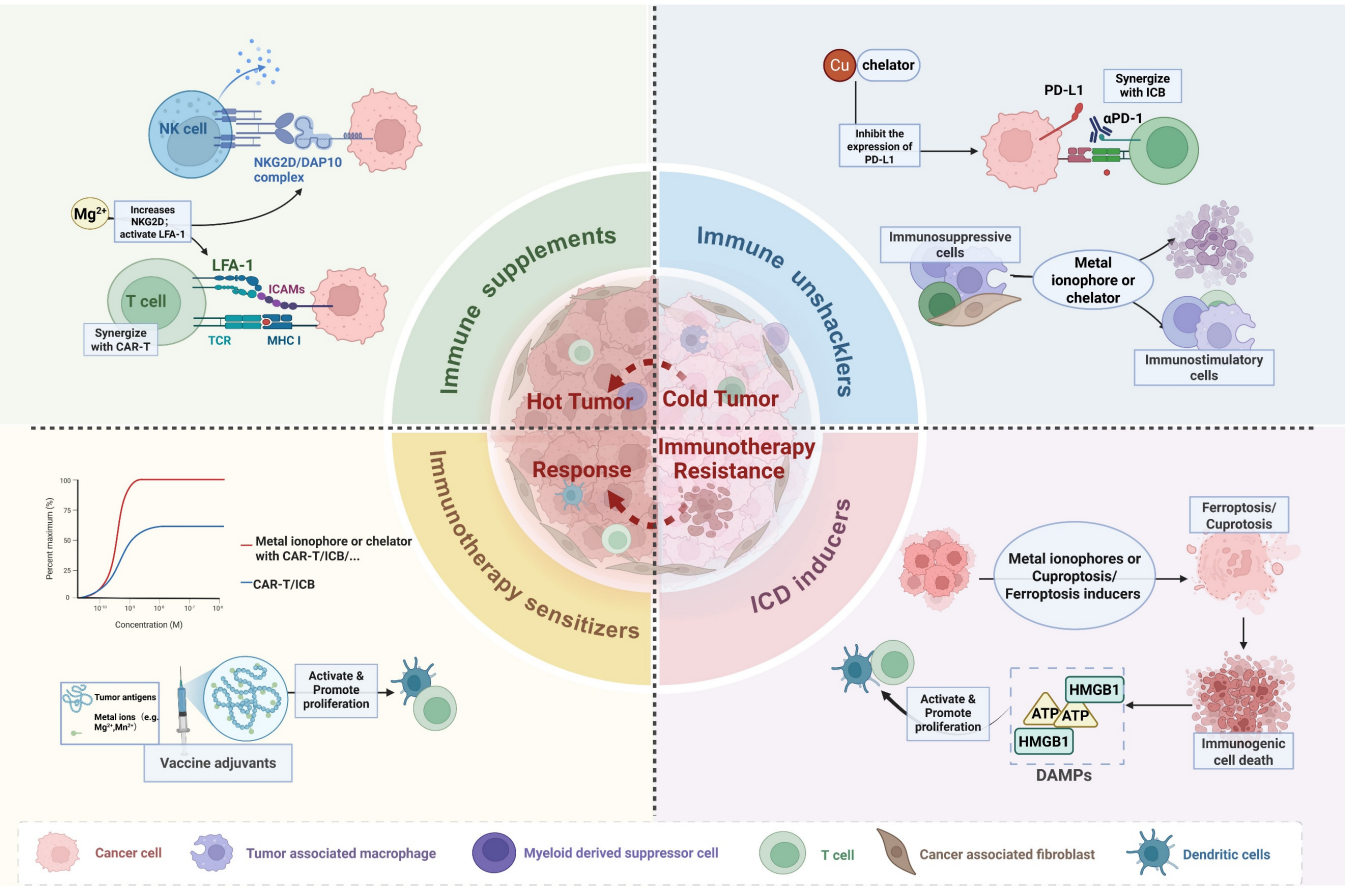
** Strategies targeting metal homeostasis imbalance for cancer treatment.** Therapeutic strategies targeting metal homeostasis imbalance in TME have significantly enhanced the efficacy and effectiveness of immunotherapy by converting immunologically "cold tumors" into "hot tumors", thereby transforming immunotherapy-resistant tumor microenvironments into immunotherapy-responsive one. Created in https://BioRender.com.

Current anti-tumor strategies targeting copper homeostasis primarily focus on two directions: (1) Copper chelators (e.g., D-penicillamine, ammonium tetrathiomolybdate (ATTM), Trientine) deplete copper in the TME, inhibiting copper-driven proliferation; (2) Copper ionophores (e.g., elesclomol, disulfiram (DSF)) elevate TME copper levels, inducing anti-tumor effects via ICD, cuproptosis and ROS burst **(Table 1, Table S1)**. Both strategies remodel the TME and show potential for synergy with immunotherapy. Nanotechnology further enhances copper-based therapeutics by enabling targeted delivery, TME-responsive release, and photothermal capabilities, creating a multimodal synergistic effect **(Figure 3)**.

#### 1.2.1 Copper chelators deplete TME copper to inhibit proliferation and remodel immunity

Copper chelators curb tumor progression by sustaining tumor dormancy, suppressing compensatory glycolysis in tumor cells, and synergizing with radio- and chemotherapy 38. More importantly, they remodel the TME to enhance immunotherapeutic synergy. This involves downregulating immune checkpoints, activating antitumor immunity, and inhibiting pro-tumor immune cell infiltration and angiogenesis.

A key mechanism of copper chelators is the inhibition of PD-L1 expression and blockade of immune escape. Current research has revealed at least three mechanisms: (1) Downregulation of JAK/STAT signaling, preventing interferon-γ (IFN-γ)-induced PD-L1 upregulation 20; (2) Suppression of EGFR signaling, promoting PD-L1 ubiquitination and degradation, thereby enhancing CD8^+^ T and natural killer (NK) cell infiltration and cytotoxicity 20; (3) Targeting phosphatidylserine (PS) on cancer cells, suppressing PD-L1 and strengthening T cell-mediated immunity 39.

Secondly, copper chelators reshape the TME into an immunologically activated state through coordinated immunomodulatory effects. For example, copper chelators (e.g., TEPA) can redirect copper ions from "copper-rich" neuroblastoma cells to "copper-poor" neutrophils. Simultaneously, copper chelators reduce TGF-β levels, upregulate KC/CXCL1, increase infiltration of neutrophils and other immune cells, and polarize neutrophils towards the pro-inflammatory N1 phenotype. This enhances their migration and ADCC function, reshapes the immunosuppressive TME, and consequently improves the efficacy of anti-GD2 antibody therapy 24. Moreover, copper chelators reduce infiltration of myeloid-derived suppressor cells (MDSCs) via Fas-FasL-mediated apoptosis 40, 41 while increasing infiltration of NK cells and CD4⁺ T cells 40, 42, redirecting low-reactive CD4⁺ T cells toward Th1-type differentiation 41 and amplifying T cell secretion of IFN-γ 40. These observations provide a theoretical foundation for the clinical translation of copper chelators.

Collectively, these multidimensional changes convert immunosuppressive niches into immunologically active microenvironments. Notably, cytokine profiling reveals compartmentalized specificity: quantitative alterations of most cytokine induced by copper chelators (e.g., IFN-γ elevation, TGF-β reduction, N1-associated factors) reach statistical significance exclusively within the TME, indicating a precisely localized immunoregulatory mechanism rather than systemic effects 24.

Nanoparticle-based copper chelators, like LDNP@mSiO₂-DPA@FA-PEG, leverage nanoscale engineering for dual therapy. Their core-shell-shell lanthanide structure (NaYF_4_:Yb,Er@NaGdF₄@NaNdF₄) provides orthogonal functions: the NaNdF₄ outer shell for efficient 808 nm photothermal conversion and the NaYF_4_:Yb,Er core for NIR-IIb fluorescence under 980 nm excitation, enabling real-time tracking and optimal photothermal therapy (PTT) timing. The resulting photothermal ablation directly kills tumors and induces ICD. Crucially, the design synchronizes therapies: PTT-triggered ICD exposes antigens while locally released DPA depletes copper to downregulate PD-L1 and inhibit angiogenesis. This engineered synergy amplifies antitumor immunity and potently inhibits metastasis, as shown preclinically 43 **(Table 5)**.

#### 1.2.2 Copper ionophores elevate copper ion levels to remodel immunity, induce ICD and trigger cuproptosis

Copper ionophores combat tumors by elevating intracellular copper levels, employing multifaceted mechanisms including ROS generation, DNA damage, suppression of tumor-associated enzymes and proteasome activity, and targeting of cancer stem cells 38. They concurrently enhance immunotherapy sensitivity through immune activation, ICD and cuproptosis triggering, and immune checkpoint molecules regulation.

In terms of mechanisms, Solier S. et al. revealed that chemically reactive copper(II) pools in mitochondria maintain NAD^+^ levels by promoting the NAD(H) redox cycle, which metabolically and epigenetically reprogramming macrophages toward the M1/pro-inflammatory state 44. This suggests that copper ionophores targeting mitochondrial copper(II) pools may facilitate the conversion of M2 macrophages to the M1 phenotype, thereby reshaping the immunosuppressive TME. Besides, Zeng et al. demonstrated in colon cancer models that copper ionophores (elesclomol/CuCl₂) establish cuproptosis-promoting conditions that suppress WNT signaling and decrease PD-L1 expression. This dual action enhances CD8⁺ T cell cytotoxicity, increases TME immune infiltration, and ultimately impedes tumor progression 45.

Secondly, copper ionophore-induced cuproptosis, ICD, and pro-inflammatory TME remodeling demonstrate profound mechanistic interdependencies. In colorectal cancer (CRC) models, cuproptosis triggered by copper ionophores provokes endoplasmic reticulum (ER) stress, driving ICD through damage-associated molecular patterns (DAMPs) release—a process that orchestrates antigen-presenting cell maturation, macrophage M1 polarization, and cytotoxic T lymphocyte activation. Notably, this cuproptosis-ICD axis is synergistically amplified when copper ionophores combine with Toll-like receptor 7 (TLR7) agonists 46.

Parallel mechanisms operate in HCC, where the copper ionophore DSF combined with copper (DSF/Cu) promotes the intranuclear accumulation and aggregation of nuclear protein localization protein 4 (NPL4), inhibits the ubiquitin-proteasome system, and induces ER stress-mediated ICD. These events collectively stimulate DCs maturation and enhance CD8⁺ T cell cytotoxicity within the TME. Particularly significant is the observed synergy between DSF/Cu-induced ICD and CD47 blockade, which overcomes immune evasion mechanisms and potentiates ICB efficacy in HCC 47.

**Figure 4:**
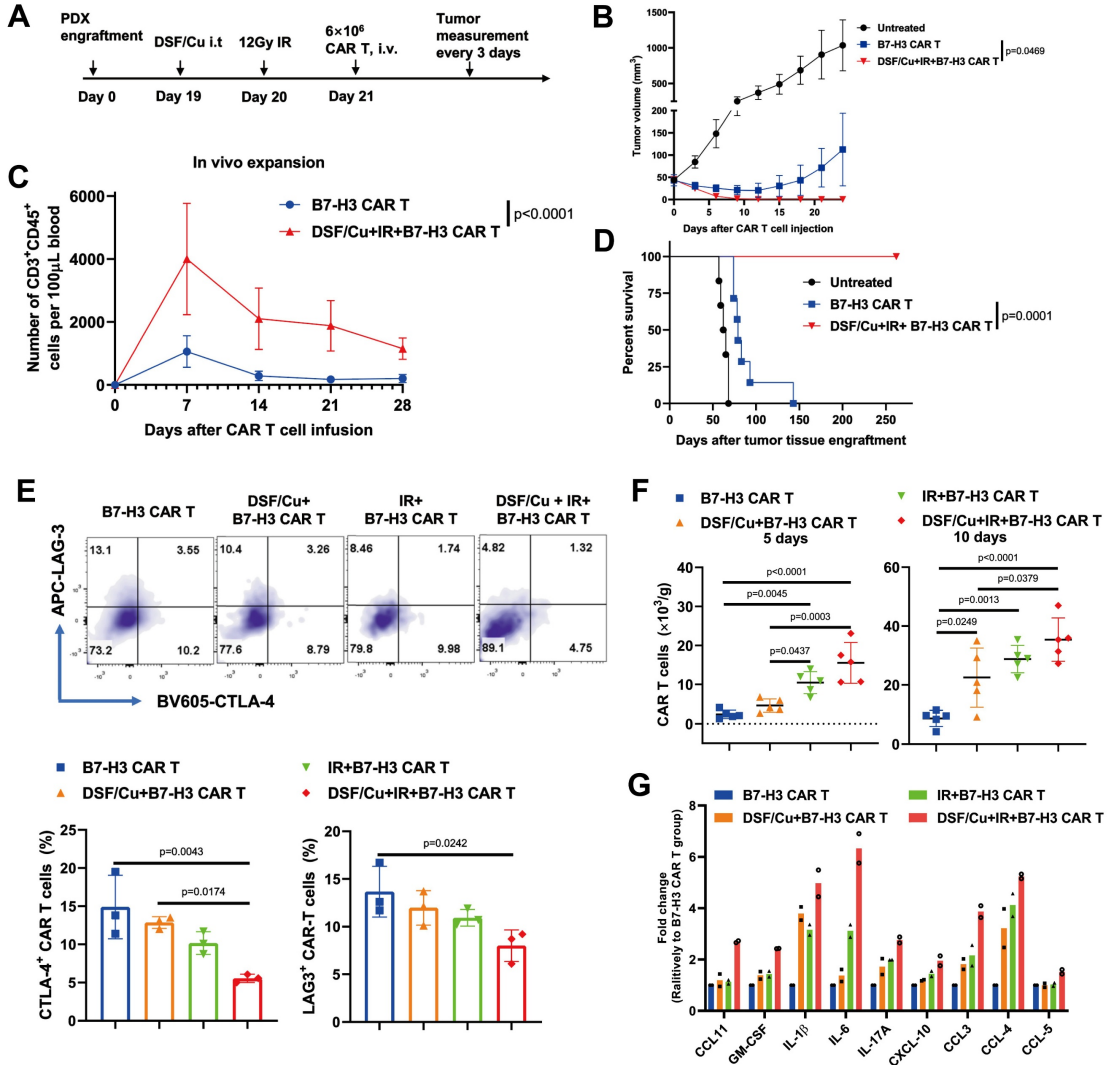
** Tumors stressed by DSF/Cu and IR reverse immunosuppressive TME and activate robust and long-sustained therapeutic responses of CAR T against solid tumors. A-D)** Therapeutic responses against solid tumors by RP B7-H3 CAR T (Reprogrammed B7-H3 Chimeric Antigen Receptor T cells) derived from PBMCs of patients with metastatic breast.** A)** Schema of TNBC-PDX (Triple-Negative Breast Cancer Patient-Derived Xenograft) mouse model. **B)** Tumor volumes (vehicle n = 6; the other two groups n = 7) of each group.** C)** Frequency of B7-H3 CAR T cells (CD3^+^ CD45^+^) in the blood of mice collected weekly (n = 7/group). **D)** Kaplan-Meier survival curve of mice (vehicle n = 6; the other two groups n = 7). **E-G)** Tumors stressed by DSF/Cu and IR reverse immunosuppressive TME in humanized mice. **E)** B7-H3 CAR T cells expressing markers associated with T-cell exhaustion (CD3^+^ CD45^+^ LAG-3^+^ or CD3^+^ CD45^+^ CTLA-4^+^) in tumor-infiltrating CAR T cells in tumor tissues 10 days after CAR T cell infusion (n = 5 mice/group). **F)** Number of B7-H3 CAR T cells in tumor tissues 5 days and 10 days after CAR T cell injection (n = 5 mice/group). **G)** Cytokine and chemokine levels in tumor tissues on day 5 after CAR T cell injection. Adapted from ref.^50^. Licensed under CC BY 4.0. Copyright 2023 The Author(s).

Furthermore, copper ionophores demonstrate significant potential for synergistic antitumor effects by sensitizing tumors to diverse immunotherapies. Mechanistically, elevated intracellular copper levels epigenetically upregulate PD-L1 membrane expression which is critical for overcoming resistance to anti-PD-L1 checkpoint blockade 20, as clinical evidence correlates low expression of PD-L1 with poor therapeutic response 48. By pharmacologically increasing copper availability, ionophores establish a targetable PD-L1-positive state, enabling previously refractory tumors to respond to immunotherapy. Single-cell transcriptomics shows that intrahepatic cholangiocarcinoma (ICC) cells susceptible to cuproptosis are typically CD274 (PD-L1)-negative and reside in a TME with reduced monocytes and macrophages. These insights have led to the proposal of combining anti-PD-L1 therapy with cuproptosis-based treatment to achieve synergistic effects in ICB treatment 49. In addition, combining disulfiram/copper (DSF/Cu) with ionizing radiation (IR) enables CAR-T cells with early memory-like properties, enhanced cytotoxicity, sustained anti-tumor responses, and improved in vivo expansion while reducing exhaustion. Mechanistically, it induces robust ICD in tumor cells, characterized by ER and oxidative stress, activation of the IRE1α-XBP1 axis, and release of DAMPs, which activates the JAK/STAT signaling pathway in CAR-T cells. Concurrently, the combination also converts the immunosuppressive TME into an immune-promoting state, significantly boosting CAR-T efficacy 50** (Table 5, Figure 4)**.

These findings above establish that copper-elevating agents functionally bridge cuproptosis, ICD induction, and immune activation, providing a compelling rationale for their synergy with existing immunotherapies. The nanoparticle NP@ESCu is designed for ROS-responsive release of elesclomol and copper ions within the TME. Its thioketal-based polymer backbone (PHPM) degrades under high ROS, triggering rapid cargo release and increasing intracellular copper by 4.2-fold. This copper overload downregulates Fe-S cluster proteins, inducing mitochondrial dysfunction and cuproptosis. RNA-seq analyses reveal activation of the cytokine-cytokine receptor interaction pathway, highlighting its immunomodulatory effects at the molecular level. Firstly, NP@ESCu induces cuproptosis in cancer cells while promoting DCs maturation and enhancing CD8⁺ T cell infiltration, thereby sensitizing them to immunotherapy. Secondly, NP@ESCu significantly upregulates PD-L1 expression on tumor cells which enhances tumor responsiveness to anti-PD-L1 checkpoint blockade, establishing potent synergy between cuproptosis induction and immunotherapy 51.

### 1.3 Current challenges and future directions

Targeting copper imbalance in the TME faces three key hurdles. First, copper biology is inherently double-edged: Copper chelators deplete TME copper to inhibit immunosuppression but may inadvertently impair cuproptosis-mediated antitumor effects. Conversely, copper ionophores induce cuproptosis yet risk exacerbating PD-L1 expression and immune evasion in resistant tumors. Second, systemic copper is tightly buffered, so prolonged manipulation may harm liver, kidney or brain. Third, tumors differ widely in cuproptosis sensitivity, making blind dosing unsafe.

Future work should therefore concentrate on precision delivery and real-time monitoring. First, utilizing nanoplatforms and subcellular mitochondrial targeting technology 52, specific copper chelators/ionophores need to be developed for precise tumor deliver, thus reducing systemic toxicity. Also, copper-64 PET 53 or activatable MRI agents can enable real-time imaging of copper flux to guide therapy and help stratify patients. Second, single-cell sequencing and high throughput screening of biopsies will identify tumors with high signatures that resist cuproptosis, allowing identifying responsive patients and personalized drug administration.

Besides, it should be mentioned that two redox-active states of copper, cuprous (Cu⁺) and cupric (Cu²⁺), within biological systems. The reversible cycling between these valence states underpins copper's role in redox reactions, contributing to both physiological enzymatic functions and pathological oxidative stress. Cu²⁺ can be reduced to Cu⁺ by cellular metalloreductases, facilitating its involvement in receptor activation and intracellular signaling. Conversely, Cu⁺ can participate in Fenton-like reactions, generating highly reactive hydroxyl radicals that promote oxidative damage to lipids, proteins, and DNA. This is a mechanism increasingly implicated in cancer progression and therapeutic resistance. In summary, the complex interconversion between Cu²⁺ and Cu⁺ and their influence on TME such as signaling pathways and redox reactions make it necessary for the design of nano-targeted drug delivery systems to take into account the issue of their valence state interconversion.

## 2. Iron

Similar to copper, iron plays a dual regulatory role in tumor biology, mediated largely by its redox activity and multiple valence states, ferrous (Fe²⁺) and ferric (Fe³⁺). On one hand, it promotes tumor proliferation and metastasis by participating in DNA replication, maintaining genomic integrity, and facilitating epigenetic regulation. More importantly, iron homeostasis dysregulation contributes to establishing an immunosuppressive TME. The redox cycling between Fe²⁺ and Fe³⁺ enables iron to participate in Fenton reactions, generating ROS that can induce ferroptosis, ICD, and oxidative stress.

### 2.1 Mechanisms of iron homeostasis dysregulation driving immunosuppressive TME

#### 2.1.1 Iron homeostasis dysregulation drives immunosuppressive TME formation

Dysregulated iron metabolism is a hallmark of both cancer cells and the TME. To support high demand for rapid proliferation, tumor cells exhibit heightened iron demand, achieving a "high intracellular iron" phenotype through mechanisms like transferrin protein (TFR1) overexpression, enhanced iron import, and suppressed iron export 54. Consequently, TFR1 serves as both a prognostic biomarker and therapeutic target 55.

Tumor cell iron sequestration within the TME creates iron-depleted conditions that alter immune cell phenotype and function. This environment fosters distinct metabolic phenotypes: cancer cells adopt an "iron-utilizing" state characterized by increased hepcidin and TFR1 expression alongside reduced ferritin levels, while lymphocytes and macrophages exhibit an "iron-donor" state marked by upregulated ferroportin 1 (FPN1) and ferritin expression, combined with elevated hepcidin and TFR1 levels 56. This indicates that immune cells may serve as iron reservoirs to sustain tumor growth, potentially impairing their own function.

Critically, iron deprivation in the TME drives tumor-associated macrophages (TAMs) polarization from pro-inflammatory M1-like to pro-tumor M2-like phenotypes. Tumor cells exploit the transferrin-transferrin receptor (TFRC) axis to competitively limit macrophage iron uptake, lowering intracellular ferrous iron. This subsequently triggers HIF-1α-mediated upregulation of M2-associated genes, promoting M2 polarization 57. M2 TAMs then adopt an "iron-releasing" phenotype by upregulating iron exporters, downregulating ferritin heavy chain 1, and enhancing heme oxygenase activity. They further supply iron to tumor cells via lipocalin 2-mediated efflux 58-60, thereby fueling tumor growth.

Conversely, increasing iron content in TAMs can reprogram them to pro-inflammatory M1-like macrophages. Research demonstrates that TAMs exposed to iron-enriched TME accumulate intracellular iron and shift toward the M1 phenotype, acquiring direct tumoricidal activity 61, 62. Notably, M1 macrophages form an "iron-sequestering phenotype" by suppressing iron exporters and activating ferritin heavy chains, thereby retaining iron within the reticuloendothelial system to maintain high intracellular ferrous iron levels 58-60.

This dynamic equilibrium underscores a key immune evasion mechanism: tumor cells induce M2 polarization by competitively depriving macrophages of iron, while exploiting M2 macrophages' iron-release function to acquire growth-essential iron, which implies that elevating TME iron may reverse this process, promoting anti-tumor M1 polarization **(Figure 1)**.

Beyond TAMs, iron also reshapes CAFs to foster immunosuppressive TME. In multiple cancers, activation of the Hmox1/iron/Kdm6b pathway transforms CAFs into "iron-loaded cancer-associated fibroblasts" (FerroCAFs). FerroCAFs recruit immunosuppressive myeloid cells via myeloid chemokines (e.g., CCL2, CSF1, CXCL1), contributing to immunosuppression and poor prognosis 63.

Taken together, these findings reveal the pivotal role of iron homeostasis dysregulation in tumor immune escape, creating a foundation for developing iron-targeted cancer treatments.

#### 2.1.2 Ferroptosis in immune cells reshapes the TME to promote tumor progression

**Figure 5:**
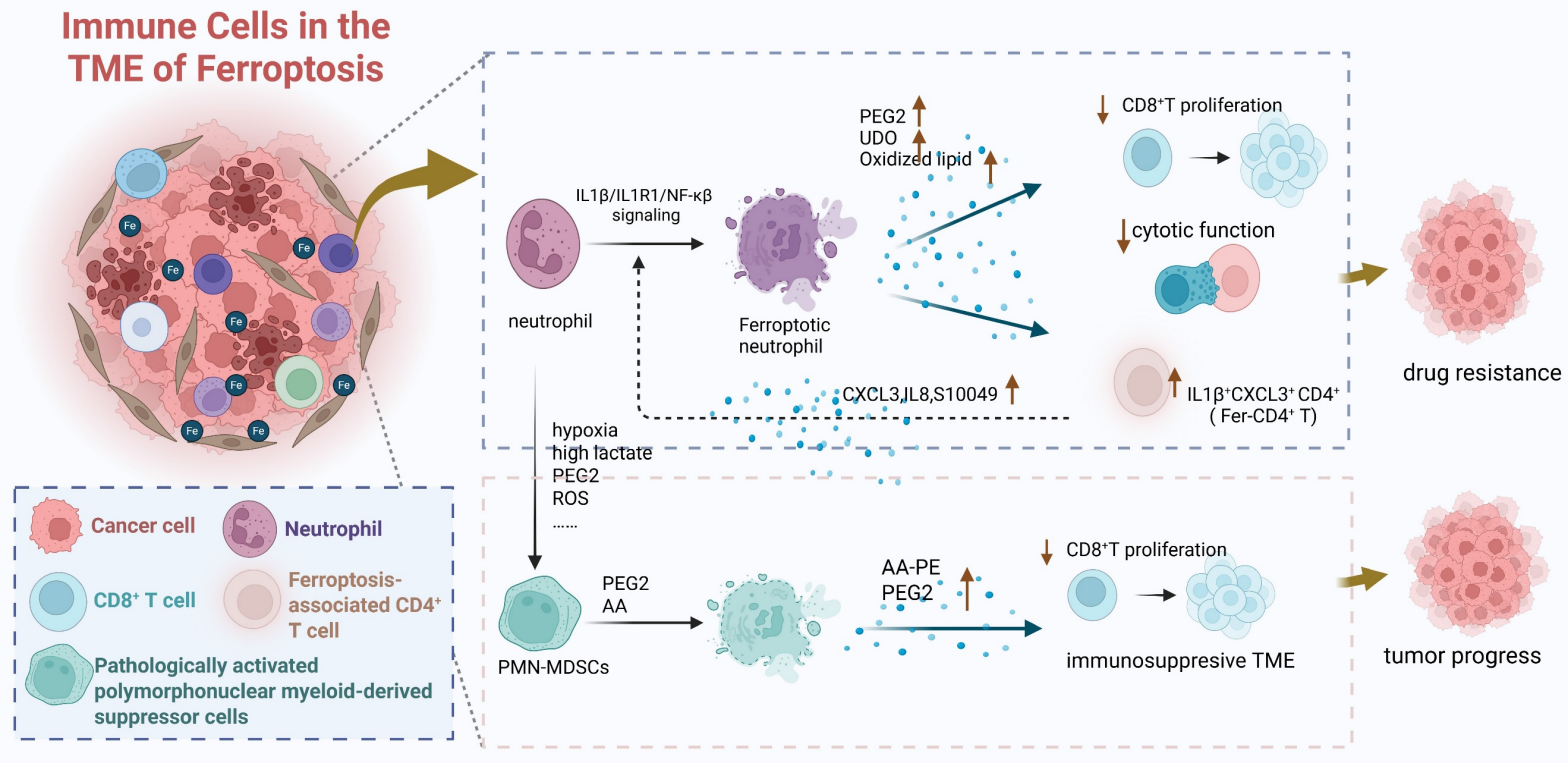
**Ferroptosis in immune cells drives the formation of TME and indirectly promotes tumor progression through the decrease in the quantity and function of immune cells and the various effective components released during ferroptosis which can affect the functions of other effector cells.** Created in https://BioRender.com.

Ferroptosis, defined by Stockwell B. R. in 2012 as an iron-dependent, non-apoptotic cell death driven by lipid peroxidation 64, can be activated within the TME by dysregulated iron metabolism in both tumor and immune cells. Ferroptosis occurring in immune cells significantly reshapes the TME into an immunosuppressive state, thereby indirectly promoting tumor progression through two interconnected mechanisms. First, ferroptosis in immune cells directly depletes anti-tumor immune cell populations and impairs their function. For instance, CAFs promote ferroptosis in NK cells by upregulating iron-inducible regulatory genes Ferroportin1 and Hephaestin and enhancing Follistatin Like 1 (FSTL1) secretion, leading to elevated free iron, upregulated NCOA4, iron-dependent lipid peroxidation in NK cells, and ultimately suppressed their anti-tumor activity 65. Similarly, in the iron-overloaded TME, ferroptosis of activated naive CD4⁺ T cells 66 and CD8⁺ T cells 67 leads to a decrease in their numbers and a decline in their anti-tumor functions. Second, the process of ferroptosis itself releases soluble mediators that actively suppress neighboring immune cells, particularly T cells **(Figure 5)**. Ferroptotic neutrophils secrete prostaglandin E2 (PGE2), Indoleamine 2,3-dioxygenase (IDO), and oxidized lipids, which inhibit CD8⁺ T cell proliferation and cytotoxicity while enriching immunosuppressive ferroptosis-associated CD4⁺ T cells (Fer-CD4⁺ T). This creates a detrimental “neutrophil ferroptosis-Fer-CD4⁺ T cell activation” positive feedback loop that drives therapy resistance 68. Analogously, pathologically activated polymorphonuclear myeloid-derived suppressor cells (PMN-MDSCs) undergo spontaneous ferroptosis, releasing oxidized lipids that potently inhibit T cell function 69. Importantly, genetic or pharmacological inhibition of ferroptosis in PMN-MDSCs reverses this immunosuppression, delays tumor progression, and synergistically enhances immunotherapy efficacy in immunocompetent mouse models 69, underscoring the therapeutic relevance of this pathway.

Multi-omics bioinformatics analyses provide strong validation for the close link between ferroptosis and an immunosuppressive TME. For example, diagnostic and prognostic models based on ferroptosis-related metabolism identify high-risk ferroptosis subgroups in HCC characterized by elevated tumor mutational burden and an immunosuppressive TME signature. This signature includes enrichment of immunosuppressive cell types (M0 macrophages, follicular helper T cells, memory B cells, neutrophils), increased expression of immune checkpoints (CD83, B7H3, OX40, CD134L), and strong associations with poor prognosis and reduced response to immunotherapy 70 **(Figure 2F-I)**. Collectively, these findings integrate data on iron metabolism regulatory networks, immune cell dynamics, and key immune molecules, highlighting the critical role for immune cell ferroptosis in driving tumor immune escape. They provide a compelling rationale for developing personalized immunotherapy strategies targeting iron metabolism pathways.

### 2.2 Strategies targeting iron homeostasis dysregulation for cancer treatment

Antitumor strategies targeting iron imbalance primarily focus on two approaches: iron depletion or ferroptosis induction. Specifically, iron depletion utilizes chelators like deferoxamine (DFO), deferiprone (DFP), and deferasirox (DFX) to reduce iron levels within the TME and tumor cells, thereby inhibiting iron-dependent proliferation. Conversely, ferroptosis induction employs agents such as system Xc⁻ inhibitors and glutathione peroxidase 4 (GPX4) inhibitors to elevate TME iron levels and combat tumors through triggering ICD, inducing ferroptosis, and causing ROS bursts **(Table 2, Figure 3, Table S1)**.

#### 2.2.1 Iron chelators block tumor iron utilization and multidimensionally activate immunity

Iron chelators inhibit tumor progression by both suppressing cellular respiration to reduce energy production 71 and inhibiting proliferation/migration signaling pathways 72. More importantly, their immunomodulatory effects significantly enhance immunotherapy synergy. Specifically, these agents remodel immune cell phenotypes, activate effector cells, and promote pro-inflammatory cytokine secretion. For instance, the intracellular chelator (TC3-S)₂ shifts macrophages from iron-releasing M2 to iron-sequestering M1 phenotypes, thereby reversing tumor growth and metastasis 73. Similarly, in ovarian cancer models, DFP-mediated iron chelation activates the cGAS-STING-IRF3 axis to drive type I interferon production, concurrently upregulating NK cell-activating death receptor 5 (DR5) expression while promoting dendritic cell-derived IL-15 secretion to sustain NK responses—collectively impeding malignant progression through multidimensional immune activation 74.

#### 2.2.2 Multiple mechanisms of ferroptosis inducers in exerting antitumor effects

Ferroptosis inducers not only directly trigger tumor cell death but also enhance immunotherapy sensitivity. Research indicates that ferroptosis is involved in T cell immunity and cancer immunotherapy. Immunotherapy-activated CD8^+^ T cells directly stimulate iron-dependent lipid peroxide accumulation in tumor cells, with amplified ferroptosis mechanism being functionally linked to improved tumor regression observed in immunotherapy protocols 75. Also, ferroptosis induction reduces MDSCs and M2-like TAMs in the TME, while enhancing the activity and infiltration of tumor-infiltrating CD4⁺ and CD8⁺ T cells 76, 77. Mechanistically, ferroptosis triggered by GPX4 inhibition leads to the exposure of calreticulin on the cell surface and release of high-mobility group box1 (HMGB1) which are key DAMPs that promote antigen presentation and myeloid cell maturation via TLR4 signaling. This shift in the immune landscape alleviates immunosuppression, thereby synergizing with ICB therapies and CXCR2 inhibition 77. Thus, targeting ferroptosis metabolism directly enhances CD8⁺ T cell-mediated tumor killing and indirectly elevates overall cancer immunotherapy response rates.

Among cancer therapies targeting ferroptosis, iron-based nanomaterials have also been extensively studied. The biomimetic magnetic nanoparticle Fe_3_O_4_-SAS@PLT, constructed from mesoporous magnetic nanoparticles (Fe_3_O_4_) loaded with sulfasalazine (SAS) and coated with platelet (PLT) membranes. The mesoporous iron oxide core affords high drug-loading capacity and pH-responsive release, while the platelet membrane cloak provides immune evasion through surface-exposed CD47 and promotes tumor accumulation via P-selectin mediated binding to CD44 receptors on cancer cells. At the mechanistic level, the released Fe²⁺/Fe³⁺ ions catalyze hydroxyl radical generation through Fenton reactions in the TME, while SAS inhibits the system Xc^‒^ transporter, depleting glutathione and synergistically amplifying lipid peroxidation, thus culminating in potent ferroptotic cell death. Beyond direct cytotoxicity, this nanoparticle induces immunogenic remodeling by promoting DCs maturation and effector T-cell recruitment. It also drives macrophage polarization from M2 toward antitumoral M1 phenotypes, thereby alleviating microenvironmental suppression and significantly enhancing the efficacy of αPD-1 checkpoint blockade therapy against metastatic tumors 78 **(Table 5)**.

Although traditional immunotherapy primarily induces regulated cell death (RCD), such death modalities are considered "immunologically silent" due to their lack of immunogenicity, making it difficult to effectively activate anti-tumor immune responses within the TME. In contrast, ferroptosis triggers ICD to amplify anti-tumor immunity. For instance, ferroptotic cancer cells promote bone marrow-derived dendritic cells (BMDCs) maturation and exert vaccine-like effects in mice, concurrently releasing ATP and HMGB1 as immune-related molecules to enhance antigen presentation 79. Furthermore, iron oxide nanoparticles (IONs) increase ICD incidence by stimulating macrophage-derived ATP and HMGB1 secretion, thereby enhancing infiltration and activation of DCs and cytotoxic T cells (CTLs) 80 **(Table 5)**. Collectively, these mechanisms provide a theoretical foundation for ferroptosis-immunotherapy synergy.

Besides, inducing ferroptosis can directly improve the efficiency and sensitivity of cancer immunotherapy. For example, the ferroptosis-inhibiting gene TYRO3 mediates resistance to anti-PD-1/PD-L1 therapy by suppressing innate immunity and ferroptosis. Conversely, inhibiting TYRO3 promotes tumor ferroptosis, sensitizing resistant tumors to αPD-1/PD-L1 therapy 81. This mechanism offers a novel therapeutic target for overcoming immunotherapy resistance.

### 2.3 Current challenges and future directions

Despite significant progress in targeting iron dysregulation for cancer therapy, two interconnected challenges hinder clinical translation. First, iron's dual impact creates a therapeutic dilemma: Iron chelation inhibits tumors but risks impeding M1 polarization 61, 62, whereas ferroptosis inducers enhance immunogenicity yet damage immune effectors 66, 67. Second, immunosuppressive cascades driven by ferroptosis (e.g., CAF-induced NK cell ferroptosis 65, neutrophil ferroptosis-Fer-CD4⁺ T cell axis 68) remain inadequately targeted, with current approaches lacking selectivity in suppressing detrimental ferroptosis and preserving immunostimulatory cell death.

Future efforts should first prioritize cell-type-specific iron modulation. This requires designing nanocarriers—such as macrophage-targeted liposomes—to deliver iron chelators or loaders with spatial precision 82, while leveraging TME-responsive materials to differentially regulate iron pools in tumor versus immune cells. Second, optimizing ferroptosis-immunotherapy synergy requires developing combinatorial regimens that exploit ferroptosis-derived immunogenic signals while blocking immunosuppressive mediators like PGE₂ and oxidized lipids 68, 69. Finally, advancing clinical biomarker stratification necessitates integrating multi-omics ferroptosis signatures with TME iron imaging to identify patients most likely to benefit from iron-centered therapies, especially those immunotherapy-resistant.

## 3. Zinc

Serum zinc levels typically decline in cancer patients, suggesting its potential as a diagnostic and prognostic biomarker 83. Zinc homeostasis imbalance is common in TMEs, exhibiting tumor heterogeneity and local specificity 84. Intratumoral zinc levels decrease in prostate, liver, pancreatic, and kidney cancers, but increase in breast, gastric, and lung cancers 85. These alterations demonstrate a close link to tumorigenesis, highlighting the crucial roles of zinc homeostasis and its regulatory proteins.

### 3.1 Mechanisms of zinc homeostasis dysregulation driving immunosuppressive TME

Like iron, zinc promotes tumorigenesis by activating transcription factors and signaling pathways involved in cancer cell proliferation and migration 86. Stromal and immune cells within the TME can act as "zinc reservoirs" for cancer cells. For instance, ZIP1-positive CAFs enhance gap junction formation in cancer cells via connexin-43 upregulation and function as Zn²⁺ reservoirs, absorbing and transferring Zn²⁺ to cancer cells, thereby promoting chemotherapy resistance in lung cancer 87.

Zinc homeostasis also directly modulates TAM phenotype and function** (Figure 1)**. In zinc-deficient HCC TMEs, the zinc transporter ZNT1 is significantly downregulated, particularly in M2 TAMs. This reduces endosomal Zn²⁺ levels, impairing the endosomal clearance of Toll-like receptor 4 (TLR4) and PD-L1 from the cell membrane. Sustained TLR4 activation increases pro-inflammatory factor release, exacerbating chronic inflammation and liver fibrosis, while surface-retained PD-L1 suppresses CD8⁺ T cell activity, facilitating immune escape and tumorigenesis. Exogenous zinc supplementation restores ZNT1 function, reduces PD-L1 expression, enhances CD8⁺ T cell cytotoxicity, and synergizes with chemo- and immunotherapy to improve efficacy 88.

Conversely, zinc-elevated TMEs can also promote immune evasion and diminish immunotherapy response through: (1) Promoting immunosuppressive phenotypes: Zinc enhances the differentiation and function of Foxp3⁺ regulatory T cells (Tregs) via FOXO1-mediated transcriptional upregulation of Foxp3 89, while simultaneously inhibiting Th1 cells, and altering CD4⁺ T cell subsets 90. (2) Upregulating immunosuppressive checkpoints: Heightened PD-1 expression on CD4⁺ T, CD8⁺ T, and γδT cells 90. (3) Reducing effector cell cytotoxicity: Decreased CD107a and lysosome-associated membrane protein 1 (LAMP1) expression on NK and CD8⁺ T cells 90. (4) Enhancing tumor anti-apoptosis: Elevated zinc in cancer cells stabilizes inhibitor of apoptosis proteins (IAPs) and inhibits caspases activation, conferring resistance to T cell-derived TNF (tumor necrosis factor)-induced apoptosis 91 **(Figure 1)**.

These findings highlight potential therapeutic targets: modulating zinc transporters; blocking STAT6/IRF4 signaling to reduce zinc flux in M2 macrophages; and antagonizing zinc-mediated anti-apoptotic pathways. Such strategies hold promises for improving the tumor immune microenvironment and enhancing therapeutic efficacy.

### 3.2 Strategies targeting zinc homeostasis dysregulation for cancer treatment

Currently, both zinc chelators and zinc ion supplements are applied to target zinc homeostasis imbalance, exerting anti-tumor effects through enhancing immunogenicity and alleviating inflammation **(Table 3, Figure 3)**.

Mechanistically, Narayan S. et al. found that zinc chelators significantly reduce Foxp3^+^ Treg cell 89. By depleting intracellular zinc in cancer cells, they rapidly degrade IAPs and restore the caspase pathway, sensitizing tumor cells to TNF-induced killing 91. Besides, zinc chelators can synergize with αPD-1 89 and CAR-T cells 91 to enhance anti-tumor effects in immunotherapy.

Conversely, zinc supplements enhance immunotherapy efficacy by reversing immunosuppressive TME. Studies have shown that intratumoral zinc injection directly induces tumor pyroptosis via both the caspase-1/GSDMD canonical pathway and the caspase-3/GSDME alternative pathway, triggered by zinc overload and ROS accumulation. This process promotes the release of pro-inflammatory cytokines (IFN-γ, IL-6, TNF-α) and increases TME infiltration of immune-promoting cells (mature DCs, CD4⁺, CD8⁺ T cells) 92 but reduces immunosuppressive cells (Tregs, MDSCs, M2 macrophages) 93. Additionally, it facilitates the endocytosis and lysosomal degradation of surface PD-L1 and TLR4 by promoting endosomal Zn²⁺ influx via zinc transporter ZNT1 in macrophages, thus dampening TLR4/NF-κB-driven inflammation and reduces PD-L1-mediated T cell suppression, and accordingly decreasing IL-6 production and enhancing CD8⁺ T cell cytotoxicity 88.

Molecularly, zinc supplementation enhances cGAS-DNA binding affinity, thereby amplifying the stimulator of interferon genes (STING)-dependent interferon response 94. Consequently, this promotes CD8⁺ T cell infiltration, activation, and effector function in the TME while suppressing tumor growth via interferon-α/β receptor (IFNAR) signaling—providing a novel strategy to boost immunotherapy response 95. Simultaneously, STING activation upregulates tumor cell MHC-I expression and antigen-presentation genes, improving tumor antigen presentation. Besides, these zinc-induced immunomodulatory effects sensitize tumors to ICB therapy, as demonstrated by the optimal CD8⁺ T cell recruitment and maximal antitumor efficacy achieved through co-administration of zinc and αPD-1 92.

In addition, leveraging zinc-based nano-drugs to chelate in situ within tumors and form toxic substances may artificially induce "zinc-induced death" in tumor cells. The TME-responsive DSF@Zn-DMSNs platform is constructed from zinc-doped dendritic mesoporous silica nanoparticles (Zn-DMSNs). This structure is further functionalized with DSF, which is loaded into the enlarged mesopores and stabilized via PEGylation. Upon exposure to the mildly acidic TME, the Zn-O bonds undergo hydrolysis, leading to biodegradation and concurrent release of Zn²⁺ and DSF, where they generate the highly toxic zinc-dithiocarbamate complex (ZnET) in situ. The highly toxic ZnET enhances autophagy while triggering ICD and facilitating CTLs infiltration. This reverses the immunosuppressive TME and achieves optimal therapeutic effects in colorectal cancer treatment 96 **(Table 5)**.

### 3.3 Current challenges and future directions

Key challenges in targeting zinc dysregulation include significant intratumoral heterogeneity and zinc's context-dependent immunomodulation: deficiency drives PD-L1 retention and CD8⁺ T-cell suppression in TAMs 88, while excess promotes Treg expansion 89 and PD-1 upregulation 90. Secondly, current systemic chelation/supplementation lacks spatial precision to resolve these dual roles.

To enable precision medicine, smart nano-delivery systems for localized zinc modulation. Further, transporters (ZIP1 in CAFs; ZNT1 in TAMs) in specific cells could be a promising target to disrupt pathological zinc flux. To overcome intratumoral heterogeneity of zinc level, integrating zinc-specific PET/MRI with zinc-relevant multi-omics signatures (e.g., transporter expression, immune cell profiling) will stratify patients for zinc-centered regimens, thereby bridging molecular imaging and targeted therapy.

## 4. Magnesium

Magnesium ions (Mg²⁺), the second most abundant cellular cation in cells, are critical cofactors and signaling molecules for normal physiology.

### 4.1 Mechanisms of magnesium homeostasis dysregulation driving immunosuppressive TME

Magnesium deficiency in the TME is associated with an increased incidence of cancers such as prostate 97 and colorectal cancer 98, as well as with poor prognosis. Magnesium deficiency promotes tumor progression by inducing genetic mutations, modulating inflammation, and activating epithelial-mesenchymal transition 98. In response, higher magnesium intake correlates with improved prognosis and reduced cancer mortality 99. Elevated serum Mg²⁺ levels were identified as an independent prognostic factor for better progression-free survival and overall survival in 1,441 ICB-treated patients within a multicenter study 100, suggesting magnesium supplementation could potentially enhance immunotherapy efficacy.

However, malignant colorectal tissues exhibit higher Mg²⁺ levels than adjacent non-cancerous tissues, and tumors with elevated magnesium content show increased mutation frequency in driver genes like TP53 and APC 98. These seemingly conflicting findings highlight the complex and context-dependent relationship between TME magnesium imbalance and tumorigenesis.

Mechanistically, TME magnesium deficiency impairs anti-tumor immunity through multiple pathways** (Figure 1)**. Firstly, low intracellular free Mg²⁺ destabilizes the critical activating receptor natural-killer group 2, member D (NKG2D) and its adaptor DAP10 in immune cells, impairing NK and CD8⁺ T cell recognition of tumor-expressed stress ligands and significantly reducing cytotoxicity. In vitro Mg²⁺ supplementation dose-dependently restores NKG2D expression and cytotoxic activity 101. Secondly, low extracellular Mg²⁺ prevents lymphocyte function-associated antigen-1 (LFA-1) integrin on T cells from adopting its active conformation, weakening LFA-1-mediated co-stimulation. This impairs tumor-specific T cell activation, effector molecule secretion, and response to ICB. Local magnesium replenishment enhances LFA-1 signaling, increasing tumor-infiltrating CD8⁺ T cells and suppressing tumor growth in mice, highlighting the therapeutic potential of targeting the Mg²⁺-LFA-1 axis 102. Thirdly, a low-Mg²⁺ TME exacerbates TAM polarization towards immunosuppression 103. As a natural blocker of N-methyl-D-aspartate receptor (NMDAR) of the macrophages, Mg²⁺ inhibits its activation by tumor-secreted glutamate, thereby countering TAM transformation into an immunosuppressive phenotype 103. Collectively, these findings underscore that restoring TME magnesium homeostasis represents a promising therapeutic strategy to enhance innate and adaptive anti-tumor immunity and ultimately improve the efficacy of cancer immunotherapies.

### 4.2 Strategies targeting magnesium homeostasis dysregulation for cancer treatment

Magnesium-targeted anti-tumor approaches focus on Mg^2+^ supplements and their derived nanomaterials. On one hand, these agents reshape the immunosuppressive microenvironment by activating immune cells. On the other hand, they directly enhance the efficacy and sensitivity of immunotherapies such as CAR-T cell function, ICB, and tumor vaccines **(Table 4, Figure 3)**.

Mechanistically, Mg²⁺ supplementation inhibits TAM immunosuppressive polarization by downregulating Arg-1, PD-L1, and vascular endothelial growth factor A (VEGFa) while upregulating pro-inflammatory cytokines and CD80/CD86 co-stimulators. This in turn promotes CD8⁺ T-cell proliferation, IFN-γ secretion, and tumor-killing capacity 103.

As an activator of the LFA-1 integrin, Mg²⁺ binding to the metal ion-dependent adhesion site promotes conformational extension and headpiece opening of LFA-1. This leads to improved calcium flux, ERK phosphorylation, metabolic reprogramming, and cytokine production in CD8⁺ T cells. Consequently, Mg²⁺ supplementation potentiates the cytotoxic function, infiltration, and immune synapse maturation of pathogen- and tumor-specific T cells. It also significantly improves CAR-T cell function 102. As noted in a previous multi-center retrospective study, serum Mg²⁺ levels are an independent prognostic factor for cancer patients undergoing ICB 100. Other studies also indicate that combining magnesium ion supplements with αPD-1 antibodies significantly increases the complete remission rate in cancer models 103. This may be related to the increased expression of PD-1 on CD8⁺ T cells following magnesium supplementation 102** (Table 5)**.

**Figure 6:**
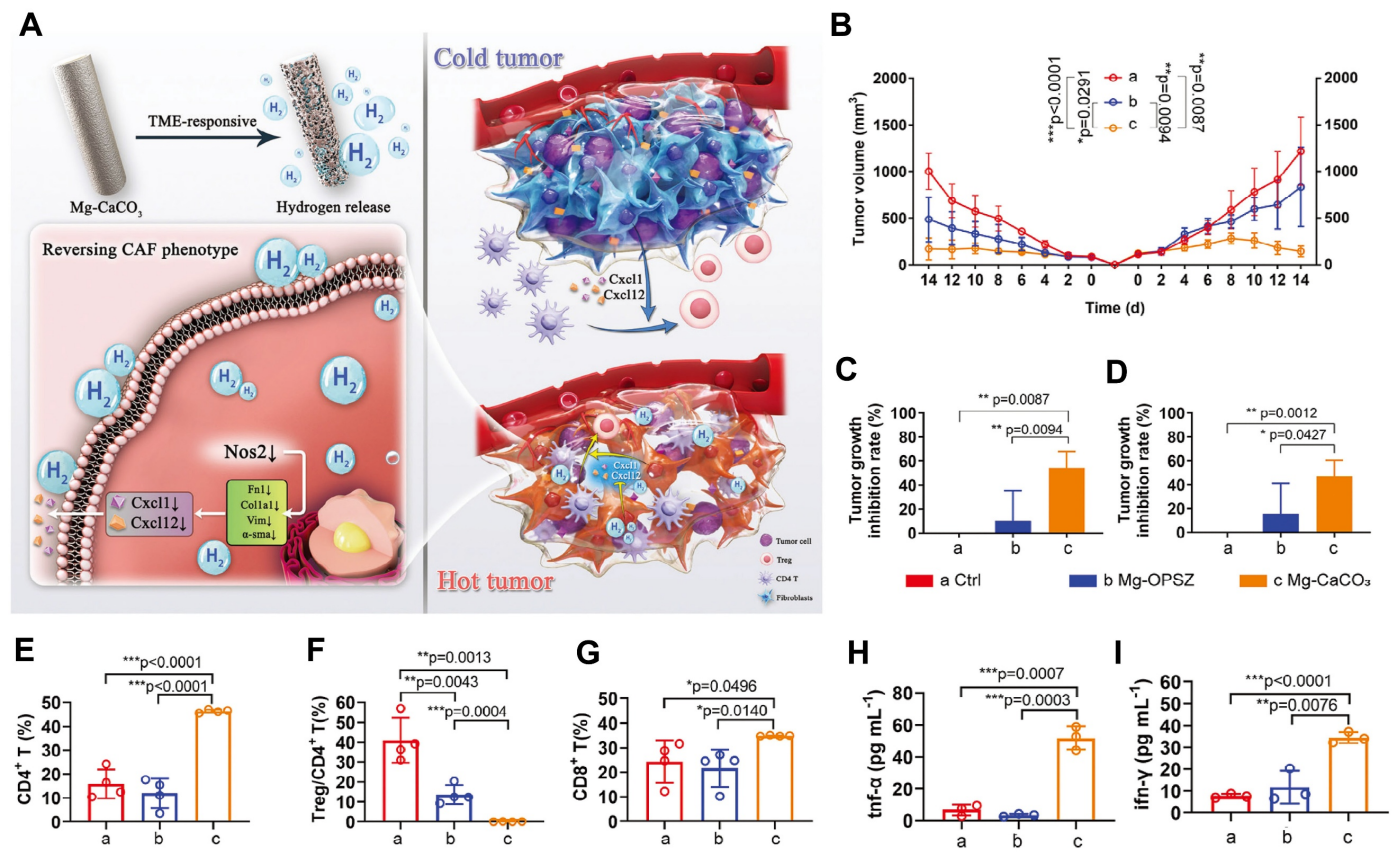
** Immunomodulatory effects of magnesium and hydrogen therapy reverse cancer-associated fibroblasts phenotypes and remodels TME to stimulate systematic anti-tumor immunity. A)** Schematic illustration of TME remodeling and tumor inhibition mechanisms of Mg-CaCO_3_. **B-I)** Systematic immune activating effects of Mg-CaCO_3_. Tumor growth curves **(B)**, tumor inhibition rate of primary tumors **(C)** and distant tumors **(D)** of Balb/c mice after different treatments (n = 5). **E-G)** Quantification of **E)** CD4^+^/CD45^+^ ratio, **F)** Treg/CD4^+^ ratio, and **G)** CD8^+^/CD45^+^ ratio (n = 4). **H-I)** Quantification of TNF-α**(H)** and IFN-γ**(I)** concentration in the serum of Balb/c mice after different treatments (n = 3). Adapted from ref.^104^. Licensed under CC BY 4.0. Copyright 2024 The Author(s).

Magnesium-based nanomaterials exert anti-tumor effects by directly inducing cytotoxicity through hydrogen generation, while synergistically enhancing immunotherapy via immunosuppressive microenvironment remodeling, antigen presentation potentiation, and T-cell activation. For example, the TME-responsive Mg-CaCO₃ system, which consists of a magnesium core coated with CaCO₃ nanoparticles embedded in an organic polysilazane matrix. This structure enables controlled hydrogen release in response to the acidic TME, where CaCO₃ dissolution exposes the Mg surface, facilitating proton-driven hydrolysis and sustained H₂ production. Functionally, the released hydrogen directly induces redox imbalance in tumor cells, leading to apoptosis and reduces ROS in CAFs, activates CD4⁺ T cells—converting "cold" to "hot" tumors 104 **(Figure 6)**. In tumor vaccine research, the R837-Pep@HM nanovaccine is composed of human serum albumin coordinated with magnesium ions and covalently conjugated with the TLR7/8 agonist imiquimod (R837) and melanoma neoantigen peptide. The human serum albumin scaffold serves as a biocompatible template that facilitates biomineralization with Mg²⁺, forming stable nanoparticles with enhanced lymphatic drainage and lymph node targeting due to optimal size and negative surface charge. The incorporation of Mg²⁺ augments DCs maturation via the Mg transporter MAGT1 and LFA-1-mediated T cell co-stimulation, while R837 activates TLR7/8 pathways, synergistically promoting neoantigen presentation and effector T-cell proliferation, thus prolonging survival in melanoma prevention models 105. These findings confirm critical role of Mg²⁺ systemic immunotherapy synergy** (Table 5)**.

### 4.3 Current challenges and future directions

Despite the established link between magnesium dysregulation and TME immunosuppression, significant challenges impede therapeutic translation. A central unresolved issue is the context-dependent duality of Mg²⁺'s role in cancer. While systemic deficiency correlates with increased cancer risk and poor prognosis, and TME deficiency demonstrably drives immunosuppression, elevated intratumoral Mg²⁺ levels have been observed in specific cancers and associated with increased oncogenic mutations. This paradox underscores the critical need to define the precise spatiotemporal dynamics and compartment-specific thresholds governing Mg²⁺'s pro- versus anti-tumor effects across different cancer types and stages. Resolving this duality is paramount to ensure the safety of Mg²⁺-modulating therapies. Secondly, achieving targeted delivery and sustained release of Mg²⁺ within the TME to maximize beneficial immune modulation while minimizing potential off-target effects remains a challenge. Furthermore, identifying robust predictive biomarkers beyond serum levels to select patients most likely to benefit from combining Mg²⁺ modulation with immunotherapies requires extensive clinical validation.

## 5. Other essential metal elements

Beyond the commonly discussed copper, iron, zinc, and magnesium, essential metals such as manganese, calcium, sodium, potassium, chromium, and molybdenum are vital for physiological functions, and their homeostasis imbalance is closely associated with tumorigenesis.

### 5.1 Mechanisms of metal homeostasis dysregulation driving immunosuppressive TME

Manganese, primarily present as Mn²⁺ in the TME, exhibits a dual role in tumor progression. First, Mn²⁺ enhances anti-tumor immunity. Manganese deficiency in melanoma, Lewis lung cancer, or T lymphoma mouse models significantly reduces TME infiltration of CD8⁺ T cells and accelerates tumor growth and metastasis 106. Conversely, Mn²⁺ acts as an integrin activator in the TME: accumulation in primary Lewis lung cancer alters tumor cell surface molecules (e.g., syndecan-1, β1-integrin) to enhance migration and invasion, while tumor-secreted Mn-containing extracellular vesicles facilitate Mn accumulation in perivascular regions and metastatic sites 107. This complex duality underscores the context-dependent impact of manganese on cancer biology.

Calcium ions (Ca²⁺), vital second messengers regulating cellular processes, significantly influence tumorigenesis, progression, and immunosuppressive TME formation. Calcium regulatory molecules mediate Ca²⁺ signaling and homeostasis, directly impacting immune cell function. For instance, deficiency of stromal interaction molecule 1 (STIM1) reduces Ca²⁺ concentration in BMDCs in mice, impairing antigen cross-presentation and migration 108. In glioblastoma, a subset of cycling tumor cells drives TME remodeling through autonomous KCa3.1-mediated calcium oscillations. These signals propagate via the tumor microtubule network, activating MAPK/NF-κB to promote proliferation and survival. Targeted KCa3.1 inhibition disrupts this extracellular Ca²⁺ "signaling hub," reduces microglial activation, and suppresses tumor growth while extending survival 109.

However, current research predominantly focuses on cancer cells rather than the TME for modulating Ca²⁺ signaling. This gap underscores the critical need to systematically investigate calcium homeostasis and its associated pathways from an integrated TME perspective, which may reveal novel therapeutic opportunities.

Beyond these, sodium (Na⁺) accumulation, a hallmark of solid tumors, contributes to an immunosuppressive TME by modulating immune cell function. High extracellular Na⁺ can induce a pro-inflammatory, yet often pro-tumorigenic, polarization state in macrophages and T cells, exacerbating tumor progression and potentially impairing cytotoxic response 110. Potassium (K⁺) shape T cell function and metabolism through membrane potential and signaling. Elevated extracellular K⁺, often resulting from tumor cell necrosis, impairs CD8⁺ T cell function by suppressing Akt-mTOR signaling and inhibiting effector responses 111.

### 5.2 Strategies targeting metal homeostasis dysregulation for cancer treatment

Mn²⁺ is a potent immune activator that enhances antitumor immunity mainly through cGAS-STING activation, improving antigen presentation and promoting CD8⁺ T cell differentiation, activation, and memory formation, along with NK cell activation and immune surveillance. The combination of Mn²⁺ with PD-1 antibodies, termed "manganese immunotherapy", significantly improves efficacy across tumor models while allowing reduced antibody doses 106. Mn²⁺ also serves as a vaccine adjuvant by enhancing antigen uptake, presentation, and germinal center formation 112. For instance, an MnO₂-based in situ vaccine (MBMA-RGD) induces ICD via photodynamic therapy (PDT) and PTT, activates STING, recruits and stimulates DCs, generates oxygen, and strongly suppresses metastasis and recurrence in mouse models without significant toxicity 113.

**Figure 7:**
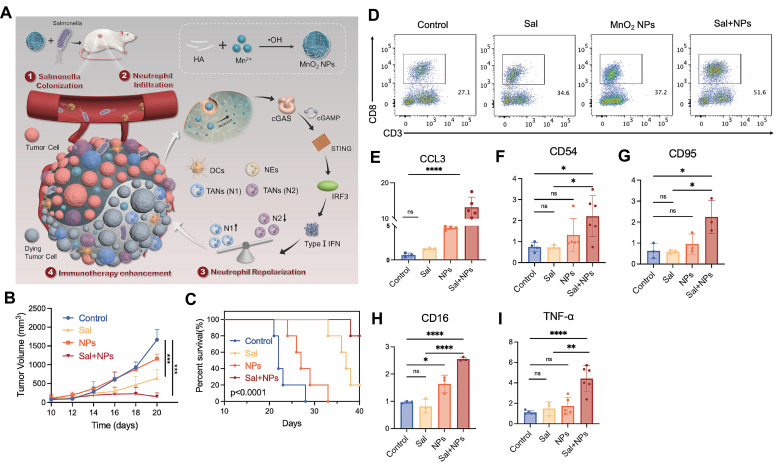
** Repolarizing neutrophils via MnO_2_ nanoparticle-activated STING pathway enhances Salmonella-mediated tumor immunotherapy. A)** Schematic illustration of the preparation of MnO_2_ NPs and the synergistic tumor therapy of salmonella and MnO_2_ NPs. **B-I)** The synergistic anti-tumor efficacy of salmonella and MnO_2_ NPs combinatorial therapy.** (B)** The tumor volume of mice and **(C)** the survival rate of mice post different treatments. **(D)** The number of CD8^+^T cells in tumor post different treatments detected by flow cytometry. **(E-I)** the level of CCL3, CD54, CD95, CD16, TNF-α in tumor tissues post different treatments detected by q-PCR. Adapted from ref. 13. Licensed under CC BY 4.0. Copyright 2024 The Author(s).

Recent advances in manganese-based nanoplatforms further demonstrate their immunomodulatory versatility. Mn-phenolic coordination networks (TMA NMs), synthesized using tannic acid and Mn²⁺ and stabilized with bovine serum albumin, disintegrate in response to pH/glutathione, releasing both Mn²⁺ and STING agonists to activate STING signaling, induce ICD, and remodel the immunosuppressive TME 114. Similarly, MnO₂ nanoparticles synergize with bacterial immunotherapies like engineered Salmonella by activating STING within the TME. Released Mn²⁺ promotes IFN-β secretion, repolarizing tumor-associated neutrophils from N2 to N1 phenotype, which reshapes the immune landscape and enhances CD8⁺ T cell infiltration and activation 13** (Figure 7)**.

Calcium-targeting strategies encompass both chelation and supplementation approaches to combat cancers. Specifically, EDTA-loaded layered double hydroxide (EDTA/LDH) nanomaterials enable acid-responsive EDTA release in tumors, depleting endoplasmic reticulum Ca²⁺ in innate immune cells. This subsequently drives anti-tumor phenotype polarization and immune cell infiltration into the TME 115. On the other hand, calcium ions and their nano-derivatives amplify ICD 116, promote M1 macrophage polarization 117, 118, and DCs maturation 117 and activate immune cells 119—thereby elevating tumor immunogenicity and potentiating anti-tumor immunity.

Furthermore, chromium ions (Cr³⁺) contribute to immune activation and synergistic immunotherapy for tumors. For example, Cr³⁺ can increase the expression of CXCL13 and CCL3 chemokines, thereby generating tertiary lymphoid structures in tumor, which promotes the infiltration, migration, and anti-tumor efficacy of CAR-T cells 120.

### 5.3 Current challenges and future directions

The therapeutic targeting of essential metals in the TME faces unresolved challenges. First, the context-dependent duality of metal actions complicates clinical translation. For instance, while Mn²⁺ bolsters cGAS-STING-mediated anti-tumor immunity 106, it concurrently enhances tumor migration and metastatic spread 107. Second, another obstacle is the limited mechanistic findings on how other essential metal reprogram the TME and the near-absence of selective drug targets. A comprehensive TME-centric understanding of metal content dynamics and associated pathways is essential to uncover novel therapeutic nodes.

To address these, future studies should develop metal-specific delivery systems to spatially restrict actions and design imaging tags that report real-time metal ion flux signals. These systems can be combined with low-dose checkpoint inhibitors to define safe windows. Furthermore, integrating single-cell metallomics will map metal-responsive immune pathways and identify targets that favor anti-tumor activity. Finally, linking these imaging readouts to accessible biomarkers will allow clinicians to titrate metal-based immunotherapies on demand.

## 6. The overlooked orchestrators: metalloproteins in metal-immunity crosstalk

While our review has focused on the immunomodulatory roles of free metal ions, it is crucial to emphasize that their biological functions are predominantly executed through a vast array of metalloproteins. These proteins, which include metalloenzymes, metallotransporters, and metal-sensing transcription factors, are the fundamental effectors that translate metal availability into cellular signals, ultimately shaping the immune landscape of the TME.

A prime example is metallothionein (MT), a family of cysteine-rich, metal-binding proteins primarily known for regulating zinc and copper homeostasis and cellular redox balance 121. MTs are not mere metal buffers; they are active players in anti-tumor immunity. In the immunosuppressive TME, MT overexpression is frequently induced by hypoxia 122 and metals themselves 123. This overexpression sequesters zinc and copper, creating a dual effect: it confers resistance to metal-induced cell death (e.g., cuproptosis) in tumor cells, promoting their survival 124, and it enlarges the redox-mobilizable metal pool, enhancing p38 MAPK activation upon stimulation and promoting Tr1 cell differentiation, which may subsequently suppress anti-tumor immunity 125. Furthermore, MTs can directly suppress the expression of pro-inflammatory cytokines and modulate the activation of NF-κB, a key transcription factor in immune responses, thereby dampening antitumor immunity 123, 126.

Beyond MTs, the zinc finger protein family, the largest class of transcription factors in humans, governs the expression of countless genes involved in immune cell differentiation and function 127. Similarly, metalloenzymes like manganese-dependent superoxide dismutase (MnSOD) and copper-containing lysyl oxidases (LOXs) are indispensable for managing oxidative stress in the TME and remodeling the extracellular matrix to facilitate immune cell infiltration or exclusion 128, 129.

The immense diversity and specificity of metalloproteins make them a rich but underexplored source of therapeutic targets. Specific targeting of metalloproteins offers promising strategies for precision metalloimmunotherapy. For example, inhibiting MT can sensitize tumors to cuproptosis or release zinc to activate T-cells. Alternatively, designing activators for metal-dependent immunoenzymes represents another exciting direction. Future research focused on mapping the "metalloproteome" of the TME will be crucial to unravel the connections between metal homeostasis, protein function, and immune regulation. These insights will ultimately reveal new nodes for therapeutic intervention.

## 7. Conclusion and perspectives

Immunotherapy has revolutionized cancer treatment, yet its efficacy remains constrained by the immunosuppressive TME. In recent years, the critical role of dysregulated metal homeostasis in shaping the immunosuppressive TME has been gradually uncovered. By regulating immune cell functions, metabolic reprogramming, and immune signaling pathway networks, it profoundly influences tumor progression and immune escape.

Metal homeostasis imbalance fundamentally shapes the immunosuppressive TME through multidimensional mechanisms. Specifically, dysregulated copper metabolism not only drives immune escape via PD-L1 upregulation and angiogenesis but induces cuproptosis and ICD to release immune-activating signals and recruit effector cells as well. Iron homeostasis dysregulation is characterized by cancer cells "hijacking" iron resources, transforming immune cells into "iron reservoirs" that polarize macrophages toward immunosuppressive M2 phenotypes and suppress the activity of NK and T cells. Notably, ferroptosis induction simultaneously kills tumor cells and releases immunogenic signals to remodel the TME into an immune-activated state. For zinc, homeostasis disruption suppresses antitumor immunity, whereas supplementation restores immune cell function, activates signaling pathways (e.g., cGAS-STING), enhances antigen presentation, and reprograms tumor immunogenicity. Similarly, magnesium deficiency impairs immune cytotoxicity, while supplementation reverses TAM phenotype, potentiates CAR-T efficacy, and synergizes with ICB. Manganese, calcium, and chromium additionally contribute indispensable TME-regulatory roles. Although there are relatively few studies on the immune regulatory effects of these elements on the TME, the potential of them and their derived nanomaterials in combination with immunotherapy is considerable but remains to be developed. For instance, manganese overcomes ICB resistance by activating the STING pathway, serves as a vaccine adjuvant, and potentiates ICD when combined with radio- or chemotherapy. Manganese-based nanomaterials further facilitate multimodal therapy through integrated drug delivery and TME remodeling 13, 114. Collectively, these findings underscore that metal homeostasis equilibrium is pivotal for sustaining antitumor immunity, thus targeting metal-immune networks offers novel therapeutic perspectives for overcoming immunotherapy resistance.

As summarized throughout this review, the intricate interplay between metal ions and immune regulation is mediated by their profound impact on key immune molecules and pathways. The regulatory relationships between the discussed metals and critical immune markers, such as PD-L1, CTLA-4, cGAS-STING, and various cytokines, are systematically cataloged in Table 6. This synthesis underscores that targeting metal homeostasis exerts its immunomodulatory effects not randomly, but through specific, molecularly definable circuits. Acknowledging these connections is therefore crucial for designing next-generation metalloimmunotherapies that precisely modulate the immune landscape of the TME.

Importantly, beyond their individual roles, a complex and synergistic crosstalk exists between different intracellular metals, forming an interconnected regulatory network that amplifies their impact on the TME. For instance, intracellular Zn²⁺ disorder can disrupt Ca²⁺ homeostasis, thereby inhibiting the electron transport chain and promoting the production of endogenous ROS, which assisted the killing of tumor cells 130. The crosstalk between zinc and copper is multifaceted. The zinc transporter ZnT1, primarily known for zinc export, also facilitates Cu²⁺ entry into cells, directly linking the homeostasis of these two metals and potentially influencing copper-dependent cell death pathways 131. Furthermore, this crosstalk can be harnessed therapeutically; as demonstrated by a recent study, a bimetallic peroxide nanoparticle co-delivering zinc and copper ions disrupts ion homeostasis to trigger PANoptosis. This process simultaneously activates multiple cell death pathways and robustly stimulates anti-tumor immune responses 132. These examples underscore that the strategic co-modulation of essential metals, rather than targeting them in isolation, can yield superior immunotherapeutic outcomes by exploiting their inherent biological interconnectedness.

The strategic modulation of essential metal homeostasis has emerged as a transformative approach in cancer therapy. While compelling evidence links specific metal imbalances within the TME to profound immunosuppression and tumor progression, the path to clinical translation is paved with both promise and complexity. There are three critical general challenges in the application of these strategies to be addressed. First, most metals exhibit low selectivity and content-dependent duality. Consequently, overload or aggressive chelation risks off-target toxicity in healthy tissues or exerts pro-tumorigenic effects, demanding precise spatiotemporal and dosage control. Second, substantial heterogeneity exists in TME metal content and homeostasis regulation mechanisms across tumor types and stages, implying that metal-homeostasis-based therapies cannot adopt a "one-size-fits-all" approach. Third, existing strategies targeting TME metal homeostasis often suffer from a dearth of validated, tumor-specific biological targets. This lack of precision hinders therapeutic specificity and efficacy, necessitating more in-depth mechanistic studies to elucidate fundamental regulatory pathways and identify novel, exploitable targets. Moreover, the clinical translation of metal-based interventions faces additional challenges related to biocompatibility, long-term safety, and potential off-target effects, which must be thoroughly evaluated in preclinical and clinical settings.

Metal-based nanomaterials represent pivotal tools to overcome immunotherapy resistance due to their precise targeting capabilities, multimodal synergistic effects, and immunomodulatory properties. Nanoparticles can effectively utilize the enhanced permeability and retention (EPR) effect of tumor tissues, passively targeting tumor sites by penetrating the loose gaps in tumor vascular endothelium while avoiding renal clearance or hepatic/splenic capture. Through surface ligand modifications, nanoparticles are endowed with specific recognition ability. Moreover, TME-responsive release (pH/GSH/enzyme-triggered) ensures spatiotemporal control. Functionally, nanoparticles realize multimodal synergistic platforms by carrying multiple therapeutic agents, which can induce ICD through treatments such as chemotherapy, radiotherapy, PDT, PTT, and sonodynamic therapy (SDT), while intrinsic metal effects remodel the TME and amplify the metal-immune cycle. In short, the spatiotemporally ordered release of metal ions precisely targets immune network interactions, while multimodal synergistic platforms pioneer novel therapeutic dimensions in antitumor immunotherapy.

Current research on metal homeostasis in the TME remains relatively under-explored compared to that on metal-based therapeutic applications. Future progress demands integrating metallomics with genomics, proteomics, and metabolomics to systematically map metal-ion binding patterns and dynamic regulatory networks in the TME. A key focus should be on decoding the interactome of different metals, such as the Zn²⁺/Ca²⁺ and ZnT1-mediated Zn²⁺/Cu²⁺ axes, to build a holistic view of the metal regulatory landscape. Concurrently, developing highly sensitive detection technologies—including single-cell metallomics, in vivo metal tracking systems, and subcellular-targeted nanoprobes—will not only elucidate the dynamic roles of metals in TME evolution but also directly guide therapeutic interventions.

In summary, the interaction between metal homeostasis imbalance and the TME defines a novel therapeutic dimension for cancer treatment. Through advancements in nanotechnology, multi-omics integration, and interdisciplinary innovation, targeting the metal-immune network holds promise for overcoming current immunotherapy limitations and revolutionizing precision oncology.

## Supplementary Material

Supplementary table.

## Figures and Tables

**Table 1:** Immunomodulatory effects of cancer treatments targeting copper dysregulation in the TME

Practical application	Functions on immunity in TME	Correlation with immunotherapy
Copper chelators	1. Inhibit PD-L1 expression in TME to suppress immune escape	Act like and synergize with ICB
	2. Modulate immune cell activity	Promote conversion to an immune-activated TME; enhance immunotherapy
	3. Elevate pro-inflammatory cytokine levels in TME	TME-specific targeting (statistically significant)
	4. Improve TME vascularization and extracellular matrix.	Facilitate immune cell infiltration and enhance immunotherapy response
Copper ionophores	1. Activate immune cells	Facilitate immune cell infiltration and enhance immunotherapy response
	2. Induce ICD via cuproptosis	Improve efficacy of TLR7 agonists and CD47 blockers
	3. Enhance sensitivity and efficiency of immunotherapies	Enhance αPD-L1 blockers and CAR-T efficacy

**Table 2:** Immunomodulatory effects of cancer treatments targeting iron dysregulation in the TME

Practical application	Functions on immunity in TME	Correlation with immunotherapy
Iron chelators	Activate immune effector cells	Promote conversion to an immune-activated TME
Ferroptosis inducers	1. Enhance innate and adaptive immunity	Synergize with ICB
	2. Induce ICD via ferroptosis	Promote conversion to an immune-activated TME
	3. Enhance sensitivity and efficiency of immunotherapies	Synergize with αPD-L1 blockers

**Table 3:** Immunomodulatory effects of cancer treatments targeting zinc dysregulation in the TME

Practical application	Functions on immunity in TME	Correlation with immunotherapy
Zinc chelators	1. Inhibit immunosuppressive cell phenotypes	Synergize with αPD-1
	2. Increase tumor cells' sensitivity to immune killing	Enhance tumor cells' sensitivity to CAR-T cell-mediated killing
Zinc supplements	1. Reverse immunosuppressive TME	Synergize with αPD-L1 blockers
	2. Activate cGAS-STING immune pathway	Conversion to an immune-activated TME; enhance the efficacy of immunotherapy

**Table 4:** Immunomodulatory effects of cancer treatments targeting magnesium dysregulation on the TME

Practical application	Functions on immunity in TME	Correlation with immunotherapy
Magnesium ion supplements	1.Reverse the immunosuppressive TME	Synergize with αPD-L1 blockers
	2.Improve sensitivity and efficiency of immunotherapies	Enhance αPD-L1 blockers and CAR-T efficacy

**Table 5:** Synergistic anti-tumor effects of metal-based nanomaterials on immunotherapy

Metal-based nanomaterials	Functions on immunity in TME	Correlation with immunotherapy	Extra functions of nanomaterials
**Nano-copper chelators**			
LMDFP 43	Inhibit immune escape by suppressing PD-L1 in TME; inhibit tumor angiogenesis	Enhance immunogenicity and immunotherapy response	PTT induces tumor ablation and ICD
RPTDH/R848 133	Inhibit tumor angiogenesis; activate/mature CAL-1	/	pH-responsive; TLR7/8 agonist (R484) delivery
**Nano-cuproptosis inducers**			
NP@ESCu 51	Induce cuproptosis in cancer cells; reverse the immunosuppressive TME; upregulate PD-L1 expression in TME	Improve αPD-L1 treatment response rate	ROS-responsive release
CAT-ecSNA-Cu 134	Reduce hypoxia in TME; enhance the sensitivity to cuproptosis; upregulate PD-L1; induce ICD via cuproptosis; activate immune response	Improve αPD-L1 treatment response rate	Loaded with catalase and immune activator
**Nano-iron-based immunomodulators**			
Ferumoxytol 135	Reverse the M2 polarization	/	/
Starch-coated iron oxide nanoparticles (IONPs) 136 ION 80	Upregulate the TLR3-TRIF-IRF3 pathway and stimulate T cells	/	/
	Promote macrophages to secrete ATP and HMGB1; enhance DCs and CTLs infiltration and activation	Enhance immunogenicity and immunotherapy response	/
**Nano-ferroptosis inducers**			
Fe_3_O_4_-SAS@PLT 78	Reverse M2 polarization; induce ferroptosis and generate mild immunogenicity	Enhance immunotherapy response	Load SAS; PLT membrane enhances circulation time and tumor targeting; pH-responsive release Redox environment-responsive; SDT based on semiconductor polymers
SINM 137	Induce ferroptosis, ICD, and polarization of M1 macrophages	Enhance immunogenicity and immunotherapy response	RSL3-loaded; PD-1 coating enhances targeting; pH-responsive release
PD-1@RSL3 NPs 138	Promote ferroptosis in tumor cells; inhibit PD-1/PD-L1; enhance CD8^+^ T cells infiltration and DCs maturation	Improve αPD-L1 treatment response rate	
**Zinc-based nanomaterials**			
DSF@Zn-DMSNs 96	Enhance autophagy; trigger ICD; facilitate CTLs infiltration	Enhance immunogenicity and immunotherapy response	pH-responsive release
**Magnesium-based nanomaterials**			
Mg-CaCO₃ 104	Reduce ROS in CAFs reshapes immunosuppressive phenotypes to activated CD4⁺ T cells	/	pH-responsive release; hydrogen therapy directly kills tumor cells
R837-Pep@HM NPs 105	Activate dendritic/T cells; enhance antigen presentation; trigger effector T proliferation	Enhance immunotherapy response	pH-responsive release; loaded with TLR7/8 agonist and melanoma neoantigen peptide
**Manganese-based nanomaterials**			
MBMA-RGD in situ vaccine 113	Induce ICD; activate STING; recruit and stimulate DCs; generate oxygen	Enhance immunogenicity and immunotherapy response	Actively targets tumor by binding integrin receptors; TME-responsive; trigger PDT and PTT
TMA NMs 114	Activate STING pathway; induce ICD	Enhance immunogenicity and immunotherapy response	pH/GSH-responsive release; loaded with STING agonist
MnO₂ NPs 13	Promote IFN-β secretion; repolarize neutrophils from N2 to N1; enhance CD8⁺ T cell infiltration and activation	Synergize with bacterial immunotherapies	/

**Table 6:** Regulation of key immune markers by essential metals in the TME

Immune marker	Category	Metal(s)	Effect	Mechanism	Ref.(s)
PD-L1	Immune checkpoint	Copper	Upregulation	Activates NF-κB, JAK/STAT, PI3K pathways; promotes immune evasion.	20, 21, 23
		Zinc	Downregulation in TAMs	ZNT1 mediates its endocytosis/lysosomal degradation in macrophages.	88
CTLA-4	Immune checkpoint	Copper	Upregulation	Positively correlates with copper transporter SLC31A1 expression.	23
TLR4	Pattern recognition receptor	Zinc	Downregulation	Zinc deficiency impairs endosomal clearance, sustaining TLR4 surface expression and chronic inflammation.	88
HIF-1α	Signaling molecule	Copper	Stabilization	Copper binding inhibits its ubiquitination, amplifying hypoxia and related immunosuppression.	28, 29, 32
cGAS-STING	Signaling pathway	Manganese, Zinc	Activation	Mn²⁺ and Zn²⁺ enhance cGAS-DNA binding, boosting IFN-I response and antitumor immunity.	106 94
NKG2D	Activating receptor	Magnesium	Stabilization	Mg²⁺ is essential for NKG2D structure and function on NK/CD8⁺ T cells.	101
LFA-1	Adhesion molecule	Magnesium	Activation	Mg²⁺ binds to LFA-1, promoting its active conformation for T cell co-stimulation.	102
Foxp3	Transcription factor	Zinc	Upregulation	Zn²⁺ promotes Treg differentiation via FOXO1-mediated transcription, enhancing immunosuppression.	89
TGF-β	Immunosuppressive cytokine	Copper	Downregulation	Copper chelators reduce TGF-β levels, alleviating immunosuppression.	24
IFN-γ	Inflammatory cytokine	Copper, Iron, Zinc, Magnesium	Upregulation	A common endpoint of enhanced T/NK cell function via metal-specific mechanisms.	40 92 103
DAMPs (HMGB1, ATP)	Immunogenic signal	Iron, Copper	Release/Upregulation	Induced by ferroptosis/cuproptosis/ICD, activating dendritic cells and T cells.	77 79

## Abbreviations

TME: tumor microenvironment
ICD: immunogenic cell death
ICB: immune checkpoint blockade
CAR-T: chimeric antigen receptor T-cell
CAFs: cancer-associated fibroblasts
GSH: glutathione
ROS: reactive oxygen species
ATP: adenosine triphosphate
HCC: hepatocellular carcinoma
PD-L1: programmed death-ligand 1
LOXL4: lysyl oxidase-like 4
ADCC: antibody-dependent cellular cytotoxicity
VEGF: vascular endothelial growth factor
ATOX1: antioxidant 1 copper chaperone
HIF-1α: hypoxia-inducible factor-1α
MT2A: metallothionein 2A
PDK1/3: pyruvate dehydrogenase kinase 1/3
DLAT: dihydrolipoamide S-acetyltransferase
DCs: dendritic cells
ATTM: ammonium tetrathiomolybdate
DSF: disulfiram
IFN-γ: interferon-γ
NK: natural killer
PS: phosphatidylserine
MDSCs: myeloid-derived suppressor cells
PTT: photothermal therapy
CRC: colorectal cancer
ER: endoplasmic reticulum
DAMPs: damage-associated molecular patterns
TLR7: toll-like receptor 7
NPL4: nuclear protein localization protein 4
ICC: intrahepatic cholangiocarcinoma
TFR1: transferrin protein
FPN1: ferroportin 1
TAMs: tumor-associated macrophages
TFRC: transferrin-transferrin receptor
FerroCAFs: iron-loaded cancer-associated fibroblasts
FSTL1: follistatin Like 1
PGE2: prostaglandin E2
IDO: indoleamine 2,3-dioxygenase
Fer-CD4^+^ T: ferroptosis-associated CD4^+^ T cell subsets
PMN-MDSCs: polymorphonuclear myeloid-derived suppressor cells
DFO: deferoxamine
DFP: deferiprone
DFX: deferasirox
GPX4: glutathione peroxidase 4
DR5: death receptor 5
HMGB1: high-mobility group box 1
SAS: sulfasalazine
PLT: platelet
RCD: regulated cell death
BMDCs: bone marrow-derived dendritic cells
IONs: iron oxide nanoparticles
CTLs: cytotoxic T cells
TLR4: toll-like receptor 4
Tregs: regulatory T cells
LAMP1: lysosome-associated membrane protein 1
IAPs: inhibitor of apoptosis proteins
TNF: tumor necrosis factor
STING: stimulator of interferon genes
IFNAR: interferon-α/β receptor
NKG2D: natural-killer group 2, member D
LFA-1: lymphocyte function-associated antigen 1
NMDAR: N-methyl-D-aspartate receptor
VEGFa: vascular endothelial growth factor A
STIM1: stromal interaction molecule 1
PDT: photodynamic therapy
MT: metallothionein
MnSOD: manganese-dependent superoxide dismutase
LOXs: lysyl oxidases
EPR: enhanced permeability and retention
SDT: sonodynamic therapy
