# Supplementary material for: Metal homeostasis as a therapeutic lever: advancing metalloimmunology to remodel the tumor microenvironment and enhance cancer immunotherapy
Source: Theranostics. 2026 Jan 1;16(3):1350–73. doi: 10.7150/thno.121988 (PMC12679368; doi:10.7150/thno.121988)
Supplement: Supplementary file 1 — Supplementary table. [file thnov16p1350s1.pdf]

Supplementary table

Table S1: Chemical properties of key metal modulating agents

Chemical name	Structure	Activation/ Metabolism	Coordination (binding) groups	Related Metal(s)
**Chelators**				
D-Penicillamine	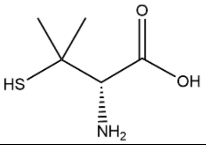	Directly active; forms stable complexes.	Thiol (-SH), Amino ($-NH_2$), Carboxylate ($-COO^-$)	Cu(I/II), Zn(II), Pb(II)
Ammonium Tetrathiomolybdate (ATTM)	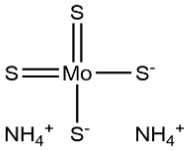	Directly active; sequesters copper in a stable tripartite complex with albumin.	Sulfide (S^{2-})	Cu(I/II)
Trientine	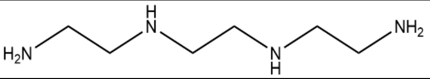	Directly active; chelates free copper.	Amino ($-NH_2$)	Cu(II)
Deferoxamine (DFO)	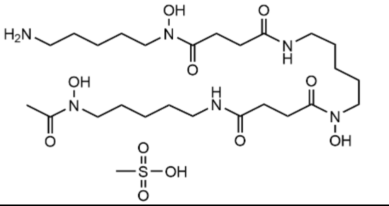	Directly active; high-affinity iron chelator.	Hydroxamate	Fe(III), Al(III), Cu(II)
Deferiprone (DFP)	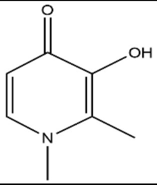	Directly active; orally active iron chelator.	Hydroxypyridinone	Fe(III), Al(III), Cu(II)
Deferasirox (DFX)	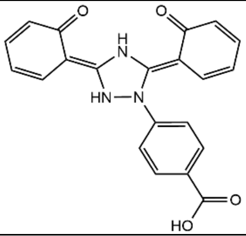	Directly active; orally active iron chelator.	Triazole / Phenolate	Fe(III)
**Ionophores**				
Elesclomol	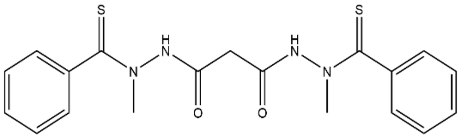	Binds extracellular Cu(II); reduced to Cu(I) upon cellular uptake, leading to cytotoxic ROS.	Thiocarbonyl (C=S), Hydrazine	Cu(II/I)
Disulfiram	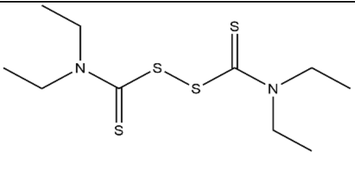	Metabolized to diethyldithiocarbamate (DDC); DDC chelates copper to form a cytotoxic complex.	Dithiocarbamate (-N(C(S)S)	Cu(II)
**Ferroptosis inducers**				

Erastin	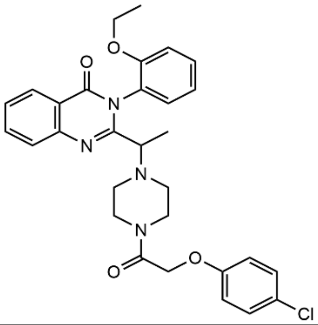	Directly active. Inhibits system xc⁻ and directly targets mitochondrial voltage-dependent anion channels.	Pharmacological inhibitor (not direct metal binding)	Pathway is Fe(II)-dependent
RSL3	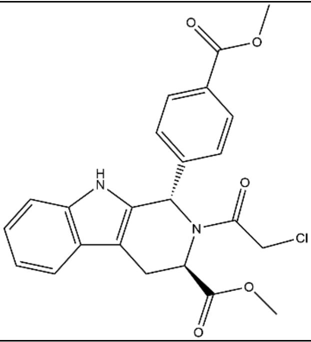	Directly active. Covalently inhibits glutathione peroxidase 4 (GPX4).	Electrophile; reacts with selenocysteine active site of GPX4	Pathway is Fe(II)-dependent
Sulfasalazine (SAS)	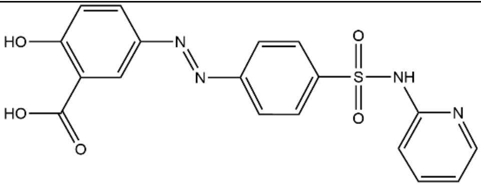	Prodrug; metabolized to 5-aminosalicylic acid and sulfapyridine. Inhibits system Xc⁻.	Pharmacological inhibitor (not direct metal binding)	Indirectly affects Fe/Cu via redox balance
